# Lipoate protein ligase B primarily recognizes the C_8_-phosphopantetheine arm of its donor substrate and weakly binds the acyl carrier protein

**DOI:** 10.1016/j.jbc.2022.102203

**Published:** 2022-06-25

**Authors:** Chetna Dhembla, Usha Yadav, Suman Kundu, Monica Sundd

**Affiliations:** 1 Department of Biochemistry, University of Delhi South Campus, New Delhi, India; 2 NMR Lab, National Institute of Immunology, New Delhi, India

**Keywords:** lipoate protein ligase B, acyl carrier protein, glycine cleavage system H protein, NMR, CoA, C_8_-CoA, lipoic acid synthesis, ACP, FAS, fatty acid synthesis, GCS, glycine cleavage system, MD, molecular dynamics, NPT, constant number of particles (N), system pressure (P) and temperature (T), NVT, constant number of particles (N), volume (V), and temperature (T), PDB, Protein Data Bank, PDC, pyruvate dehydrogenase complex, RMSF, root mean square fluctuation, SPR, surface plasmon resonance, STD, saturation transfer difference

## Abstract

Lipoic acid is a sulfur-containing cofactor indispensable for the function of several metabolic enzymes. In microorganisms, lipoic acid can be salvaged from the surroundings by lipoate protein ligase A (LplA), an ATP-dependent enzyme. Alternatively, it can be synthesized by the sequential actions of lipoate protein ligase B (LipB) and lipoyl synthase (LipA). LipB takes up the octanoyl chain from C_8_-acyl carrier protein (C_8_-ACP), a byproduct of the type II fatty acid synthesis pathway, and transfers it to a conserved lysine of the lipoyl domain of a dehydrogenase. However, the molecular basis of its substrate recognition is still not fully understood. Using *Escherichia coli* LipB as a model enzyme, we show here that the octanoyl-transferase mainly recognizes the 4′-phosphopantetheine-tethered acyl-chain of its donor substrate and weakly binds the apo-acyl carrier protein. We demonstrate LipB can accept octanoate from its own ACP and noncognate ACPs, as well as C_8_-CoA. Furthermore, our ^1^H saturation transfer difference and ^31^P NMR studies demonstrate the binding of adenosine, as well as the phosphopantetheine arm of CoA to LipB, akin to binding to LplA. Finally, we show a conserved ^71^RGG^73^ loop, analogous to the lipoate-binding loop of LplA, is required for full LipB activity. Collectively, our studies highlight commonalities between LipB and LplA in their mechanism of substrate recognition. This knowledge could be of significance in the treatment of mitochondrial fatty acid synthesis related disorders.

Lipoic acid is a hydrophobic cofactor, essential for the function of several metabolic enzyme complexes, *viz.* pyruvate dehydrogenase complex (PDC) required for pyruvate oxidation, 2-oxoglutarate dehydrogenase as a component of Krebs cycle (OGDHc), glycine cleavage system (GCS) involved in glycine degradation, branched-chain keto acid dehydrogenases (BCKDHc) necessary for the metabolism of branched chain amino acids, and 2-oxoadipate dehydrogenase in lysine metabolism (1, 2, 3, 4, 5). These are multimeric enzymes with a conserved lipoyl domain that serves as a substrate for lipoic acid modification. Lipoyl lysine acts as a “swinging arm,” helping to shuttle intermediates between the active site of multienzyme complexes (1, 4, 6, 7, 8, 9). The flexibility of lipoic acid is crucial for substrate channeling and electron transfer during oxidation–reduction reactions (10).

Most of the knowledge with regard to lipoic acid metabolism comes from studies conducted on *Escherichia coli*, *Listeria monocytogenes*, *Bacillus subtilis*, and *Staphylococcus aureus* (11). In the presence of free lipoate or octanoate, lipoic acid is salvaged from the environment by lipoate protein ligase A (LplA) (12, 13, 14). An activated lipoyl-5′-AMP intermediate is formed from ATP and lipoic acid on the surface of LplA. Thereafter, the ε-amino group of a lipoyl lysine attacks the noncovalently bound lipoyl-AMP, forming an amide linkage with lipoyl-group and releasing AMP (3, 8, 9, 14, 15). All LplA molecules comprise a large N-terminal catalytic domain (lipoic acid binding) and a small C-terminal domain (16). In lipoic acid–deficient environments, lipoate protein ligase B (LipB) also known as Lipoyl-octanoyl transferase catalyzes the first biosynthetic step of lipoic acid synthesis (4). Studies on *Mycobacterium tuberculosis* (Protein Data Bank [PDB] 1W66) and *Thermus thermophilus* (PDB 2QHS, 2QHT, 2QHU, and 2QHV) LipB suggest conservation of a structural scaffold, similar to the N-terminal catalytic domain of LplA. LipB functions as a cysteine/lysine dyad acyltransferase, cysteine 176 and Lys 142 of *M. tuberculosis* LipB (Cys169 and Lys 135 of *E. coli* LipB) functioning as acid/base catalysts (17, 18, 19). A covalent octanoyl-LipB thioester intermediate is formed by the transfer of octanoyl-chain from C_8_-ACP (acyl carrier protein) to Cys176 of LipB (20). Subsequently, the thioester bond is attacked by the ε-amino group of the lipoyl lysine, resulting in C_8_- modification of the latter. In the following step, octanoyl group covalently tethered to the lipoyl domain is converted to lipoic acid in the presence of LipA (lipoyl synthase) by insertion of two sulfur atoms.

In metazoans, three different enzymes participate in lipoic acid synthesis: (a) lipoyl transferase 2 (LipT2) that transfers octanoyl chain from C_8_-ACP to the lipoyl subunit of GCS, (b) lipoic acid synthase (LIAS), a sulfur insertion enzyme that adds two sulfur atoms to the C_8_- chain to form lipoic acid, and (c) LipT1, an amidotransferase, that relays octanoyl-chain/lipoic acid from the H subunit of GCS to the lipoyl carrier protein subunit of other dehydrogenases, *viz.* PDH, BCKDHc, αKGDH. Malfunction of any of the gene products causes lipoic acid synthesis defects in neonatal patients, underscoring the need to understand the pathway at the molecular level. Disorders range from defective mitochondrial energy metabolism, toxic levels of certain amino acids, neurological problems, and even death, depending on which lipoic acid synthesis gene malfunctions (11, 21, 22). Similar clinical phenotypes are also observed if the upstream mitochondrial fatty acid synthesis (FAS) that does not function properly, for example, mitochondrial enoyl-CoA reductase associated disorder (MECR) (21, 23), malonyl-CoA synthetase (ACSF3), malonyl CoA-acyl carrier protein transacylase (MCAT), etc. (24). Defects in GCS gene also causes elevated glycine levels in the brain, leading to analogous neurological problems (25, 26). Impaired dehydrogenase activity causes respiratory defects and muscle weakness due to the disruption of Krebs cycle (5, 12, 13).

Interestingly, all lipoic acid metabolism enzymes belong to the Pfam PF03099 family and share a similar scaffold, despite low sequence conservation. They differ remarkably in their catalytic mechanism and interactions (20, 27). Reasonable biochemical/structural data exist for the free, as well as substrate-bound forms of LplA from *E. coli*, *Thermoplasma acidophilum*, *Enterococcus faecalis*, *Streptococcus pneumoniae*, and *Mycoplasma hypopneumoniae*. However, the biosynthetic enzyme LipB is relatively less explored. Structures are available from only two sources, *M. tuberculosis* and *T. thermophilus* for this enzyme. The mechanistic understanding of LipB interaction with its substrates is also sparse. Two recent studies on LipB have highlighted its ability to allosterically distinguish between different acyl-ACP's, *viz.* C_6_-, C_8_-, C_10_-, and C_12_-ACP based on differences in their surface conformation (28, 29). Our work extends their findings one step further and shows that the enzyme can differentiate between apo-, holo-, and C_8_-ACP as well. The enzyme binds C_8_- and holo-ACP with moderate affinity but weakly associates with apo-ACP. It can uptake octanoate from its cognate C_8_-ACP, noncognate C_8_-ACP's, as well as octanoyl-CoA. Chemical shift perturbation studies support the binding of adenosine and phosphopantetheine arm of CoA with LipB. Interestingly, mice LipT2 also successfully transferred octanoate-CoA from C_8_-CoA to *E. coli* GcvH *in vitro*, suggesting a common mechanism of substrate recognition by most octanoyl transferases.

## Results

LipB (UNP60720) catalyzes a bisubstrate reaction, using C_8_-ACP (molecular weight 9.4 kDa) as a donor substrate and lipoyl domain of dehydrogenases, for example, GCS H protein (GcvH), PDC, or 2-oxoglutarate dehydrogenase complex (OGDH) as octanoate acceptors (25). Limited structural/biochemical data exist for the LipB–ACP complex, highlighting the need for in-depth studies. In the absence of an *E. coli* LipB structure, *M. tuberculosis* LipB 1.08 Å crystal structure (*Mt*LipB, PDB 1W66) has been used as a structural model to interpret our findings.

**Figure 1:**
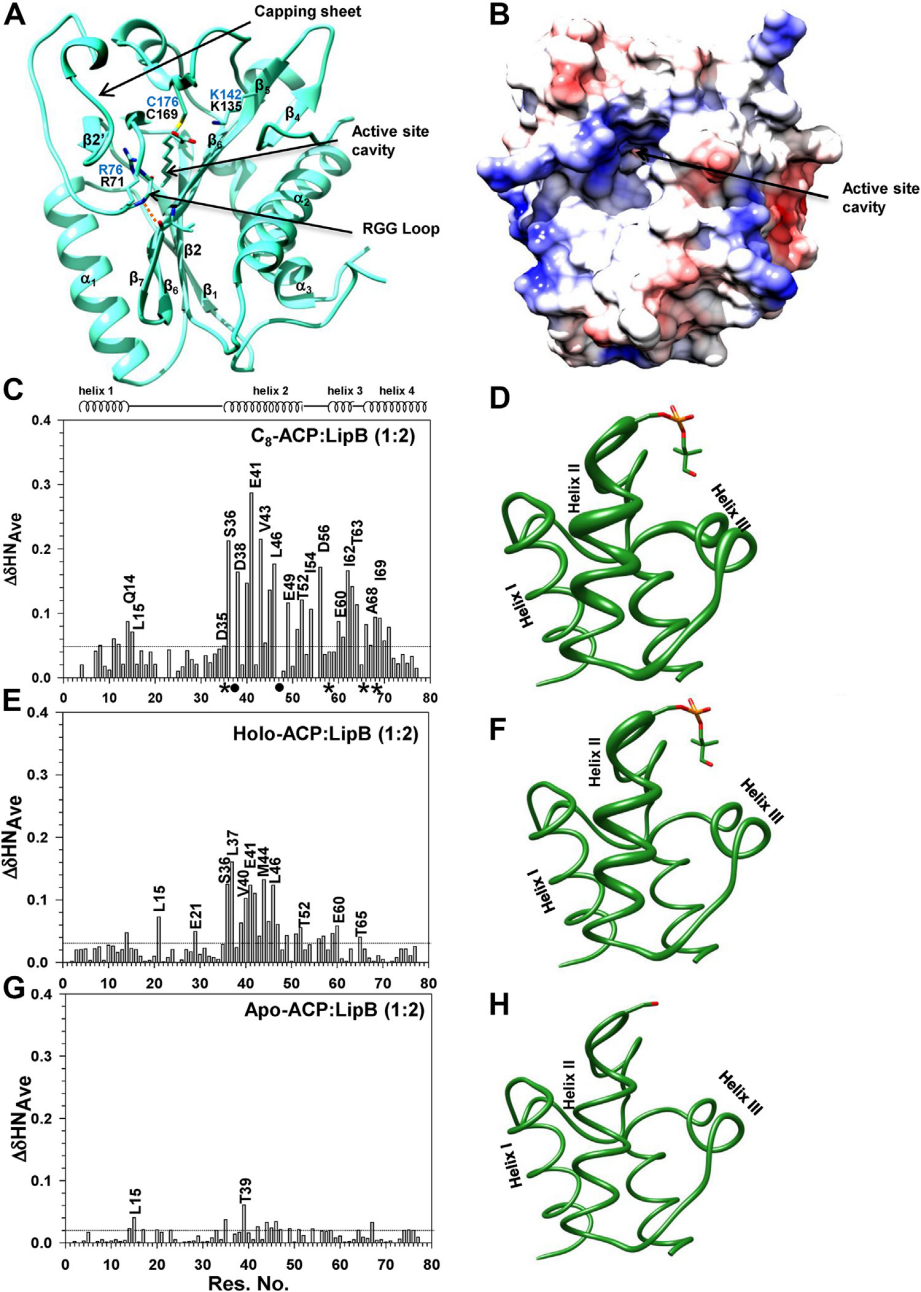
**Backbone chemical shift perturbations of *E. coli* ACP upon LipB interaction.***A*, ribbon representation of *M. tuberculosis* LipB (PDB 1W66) covalently bound to a decanoyl-chain. The positively charged residues in the active site cavity are shown as *sticks*. *M. tuberculosis* residue numbering is shown in *blue*, and the corresponding *E. coli* numbering in *black*. *B*, a surface representation of *M. tuberculosis* LipB (colored based on coulombic charge), displaying the opening of the active site cavity. Changes in the amide chemical shift of (*C*) C_8_-ACP, (*E*) holo-ACP, and (*G*) apo-ACP, upon titration with unlabeled LipBK135A/C169A. ACP backbone has been represented as a worm (PDB 5VCB, chain D) for (*D*) C_8_-ACP, (*F*) holo-ACP, and (*H*) apo-ACP displaying chemical shift changes upon LipBK135A/C169A interaction. The magnitude of chemical shift change at each amide is directly proportional to the thickness of the worm. PDB, Protein Data Bank.

*M. tuberculosis* LipB comprises five central β-strands ↑β_1_,↑β_2_,↓β_3_,↑β_7_,↓β_6,_ enclosed by three long helices, α_2_ and α_3_ on one face and α_1_ on the other side of β sheet, illustrated in Figure 1*A*. Some of the catalytically important residues of *M. tuberculosis* LipB are shown as *sticks* and labeled *cyan*. The corresponding *E. coli* LipB residues are also labeled *black*. A short stretch of beta strands comprising ↓β2',↓β1',↑β3', called the 'capping sheet', is also present in LipB, of unknown function. Figure 1*B* shows the molecular surface of *Mt*LipB, colored based on coulombic charge, displaying the position of active site cavity. *E. coli* LipB amino acid sequence has been compared with other octanoyl transferases in Fig. S1. *E. coli* LipB (UNP 60720) shares 32% sequence identity with *M. tuberculosis* LipB (UNP P9WK83, PDB 1W66), 28% with human mitochondrial LipT2 (UNP A6NK58), 29% with *Mus musculus* LipT2, and 26% with *Leishmania major* enzyme (UNP Q4A1B1).

### LipB binds octanoyl-ACP with moderate affinity

To gain insights into the LipB:C_8_-ACP interaction, a catalytically inactive *E. coli* LipBK135A/C169A double mutant was prepared, and its interaction with ^15^N labeled *E. coli* C_8_-ACP followed in a ^1^H^15^N transverse relaxation optimized spectroscopy (TROSY)–heteronuclear single quantum coherence (HSQC) NMR experiment. C_8_-ACP was stable at room temperature for approximately 7 days, as shown in Fig. S2. The protein did not display any hydrolysis during this time, confirming its suitability for NMR studies. ACP backbone was assigned based on the BMRB ID 27061, and standard 3D NMR experiments were acquired to assign the octanoyl-ACP spectra. To give equal weights to proton and nitrogen chemical shifts, changes upon LipB interaction have been reported as their weighted average, determined using Equation 1.

*E. coli* ACP displays a four-helix bundle fold, consisting of helix I (Thr 2-Gly 16), helix II (Asp 35-Asp 51), helix III (Asp 56-Thr 64), and helix IV (Thr 64-His 75). Titration with LipB resulted in perturbation of several C_8_-ACP backbone amides *viz.* Gln 14, Leu 15, Asp 35, Ser 36, Asp 38, Val 40, Glu 41, Val 43-Leu 46, Glu 49, Thr 52, Ile 54, Asp 56, Glu 60-Thr 64, Glu 66, and Ala 68-Tyr71 (Fig. 1*C*). These residues are present in helix II, III, and IV of ACP. The peaks for Asp 35, Glu 57, Val 65, and Ala 68 were broadened beyond 1:1 ACP:LipBK135A/C169A ratio, shown by a (∗) in the figure. Leu 37 and Glu 48 displayed chemical exchange line broadening right from the first titration point, shown as (•) in the figure. One SD has been used to demarcate significant chemical shift change. Perturbations have been mapped to the *E. coli* holo-ACP structure (PDB 5VCB, chain D) represented as a worm in Figure 1*D*. Thickness of the worm is directly proportional to the magnitude of chemical shift change. Fig. S3*A* shows the multiple overlaid ^1^H^15^N TROSY-HSQC spectra for C_8_-ACP:LipBK135A/C169A titration. Each titration point is shown in a different color: free C_8_-ACP is colored *red*; C_8_-ACP:LipBK135A/C169A 1:0.25 M ratio *blue*; 1:0.5 *magenta*, 1:0.75 *cyan*, 1:1 *green*, 1:1.5 *orange*, and 1:2 *purple*. Same color coding has been used throughout for all the titrations reported in this study.

The binding affinity of LipBK135A/C169A for C_8_-ACP was determined by surface plasmon resonance (SPR) measurements, immobilizing LipBK135A/C169A on the surface of a sensor chip, and passing increasing concentrations of C_8_-ACP over the chip surface. Fig. S4*A* shows the SPR sensorgram and Fig. S4*B* binding curve for C_8_-ACP:LipBK135A/C169A interaction. The two proteins interact in micromolar range, with a dissociation equilibrium constant (*K*_*D*_) of 36.4 ± 0.19 μM.

### LipB binds holo-ACP with relatively lower affinity

Holo-ACP contains a phosphopantetheine modification akin to C_8_-ACP, but lacks the octanoate group. The contribution of C_8_-chain in the interaction of C_8_-ACP with LipB was also investigated. ^15^N-labeled holo-ACP (containing ^15^N-labeled 4′-phosphopantetheine arm) was titrated with unlabeled LipBK135A/C169A. Noticeable changes in the amide chemical shift were observed for several holo-ACP residues. As shown in Figure 1*E*, Gln 14, Glu 21, Val 29, Ser 36, Leu 37, Thr 39, Val 40, Glu 41, Leu 42, Val 43, Met 44, Ala 45, Leu 46, Glu 47, Asp 51, Thr 52, Glu 53, Ile 54, Glu 57, Ala 59, and Glu 60 displayed significant perturbations. The aforementioned residues are present in helix I, loop I, helix II and helix III of ACP, shown as a worm in Figure 1*F*. Fig. S3*B* displays multiple overlaid ^1^H^15^N TROSY-HSQC spectra for holo-ACP upon titration with LipBK135A/C169A. The direction of chemical shift change with increasing LipBK135A/C169A concentration is shown by a forward pointing *arrow*. Interestingly, one of the two NH resonances of 4′-phosphopantetheine moiety (^15^N-labeled phosphopantetheine expressed in holo-form in *E. coli*) observed at a chemical shift of 8.1 ppm in the ^1^H^15^N TROSY-HSQC spectra also displayed significant change during the titration (Fig. S3*D*).

Fig. S4*C* shows the SPR sensorgram, and Fig. S4*D* shows binding curve for the interaction of LipBK135A/C169A with holo-ACP. The binding curve did not reach saturation, and therefore, the estimate of affinity has been reported as apparent binding constant. A *K*_*D*_^app^ of 100 ± 0.43 μM was observed for the LipB:holo-ACP interaction.

### apo-ACP weakly interacts with LipB

In the cell, ACP is expressed as an apo-protein and posttranslationally modified to holo-ACP by the catalytic action of 4′-phosphopantetheinyl transferase. The importance of 4′-phosphopantetheine arm in the interaction of LipB with its substrate was also probed. ^1^H^15^N apo-ACP was titrated with unlabeled LipBK135A/C169A (Fig. 1*G*). Insignificant changes in the amide chemical shift were observed (<0.06 ppm) upon addition of up to 2 M equivalents of LipBK135A/C169A. In the superimposed spectra, most of the amide resonances of apo-ACP overlapped with the peaks in presence of LipBK135A/C169A (1:2 M ratio, Fig. S3*C*). A worm figure of apo-ACP is also shown, displaying insignificant changes upon interaction with LipBK135A/C169A (Fig. 1*H*).

SPR measurements were also performed to understand the strength of this interaction. Relatively higher concentration of apo-ACP was required, compared to C_8_- and holo-ACP titrations. Fig. S4*E* shows the SPR sensorgram, and Fig. S4*F* shows binding curve for the interaction of LipBK135A/C169A with apo-ACP. A *K*_*D*_^app^ of 2.1 ± 0.18 mM was obtained for the binding interaction.

### Noncognate ACPs can serve as a donor substrate for LipB

**Figure 2:**
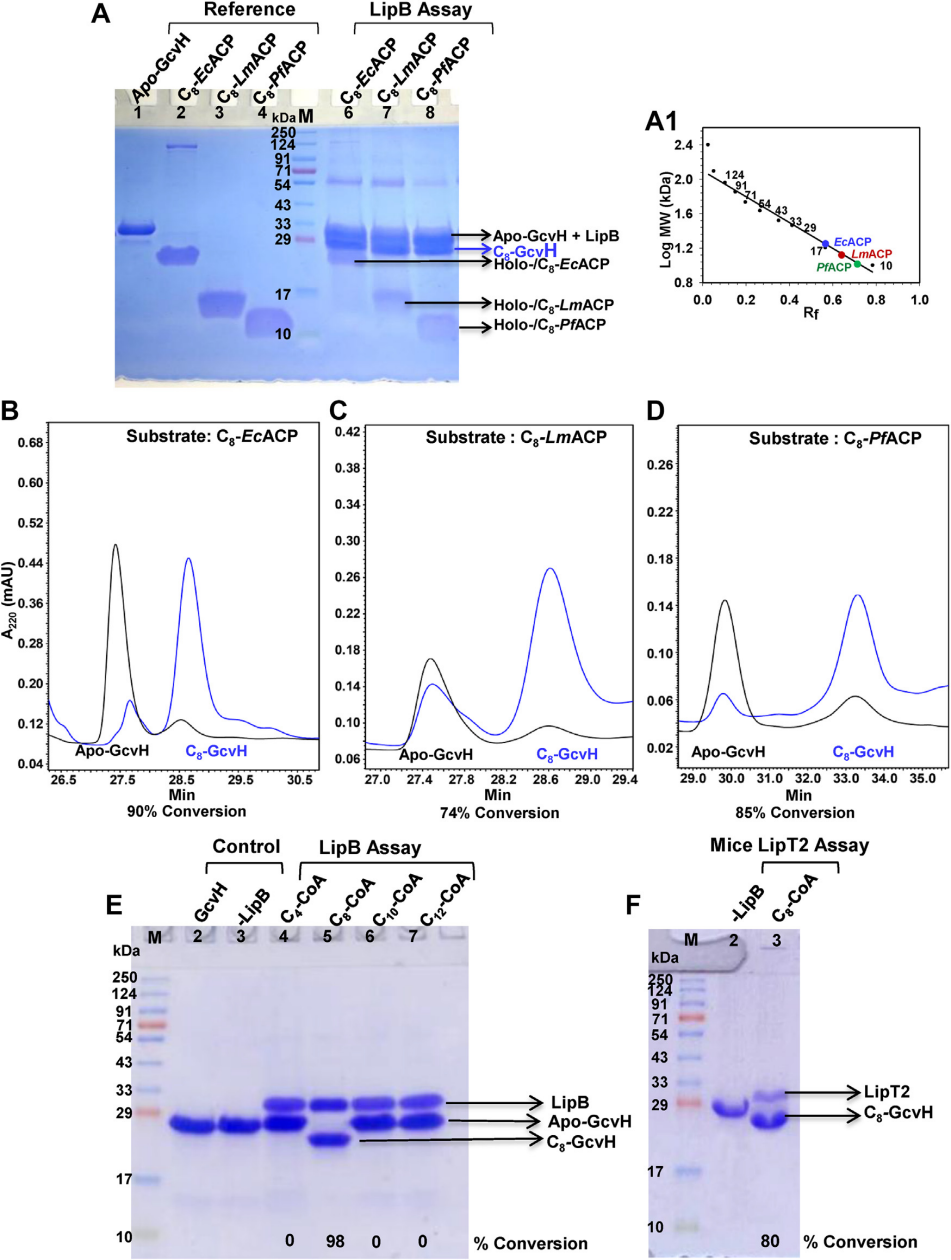
**LipB can use noncognate ACP as well as C**_**8**_**-CoA as octanoate donor.***A*, a 12% SDS-PAGE gel displaying lane 1: Apo-GcvH, lanes 2 to 4: C_8_-ACP, C_8_-*Lm*ACP, and C_8_-*Pf*ACP loaded for reference, lane 5 molecular weight marker, lane 6 to 8: LipB assay using C_8_-ACP (*E. coli*), C_8_-*Lm*ACP (*L. major*), and C_8_-*Pf*ACP (*P. falciparum*), respectively, as substrates. *A*1, a standard curve prepared based on the migration of standards in the SDS-PAGE gel of (*A*). The migration of *E. coli* ACP corresponds to a molecular weight of 18 kDa, *Lm*ACP ∼14 kDa, and *Pf*ACP ∼12 kDa. C18-reversed phase chromatogram for the GcvH conversion after the LipB assay performed using (*B*) C_8_-*Ec*ACP, (*C*) C_8_-*Lm*ACP, and (*D*) C_8_-*Pf*ACP, as octanoate donors. The chromatogram before the assay is shown in *black* and after the assay in *blue*. %GcvH conversion is shown at the *bottom* of each chromatogram. *E*, a 12% SDS-PAGE gel displaying the conversion of GcvH, using different acyl-CoA as octanoate donors. Lane 2: apo-GcvH, Lane 3: assay in the absence of LipB, Lane 4 to 7: assay in presence of C_4_-CoA, C_8_-CoA, C_10_-CoA, and C_12_-CoA, respectively. *F*, a 12% SDS-PAGE gel, displaying the conversion of *E. coli* GcvH in presence of *Mus musculus* LipT2, using C_8_-CoA as octanoyl donor. Lane 1: molecular weight marker, lane 2: apo-GcvH, lane 3: assay in the presence of mice LipT2. % conversion determined using HPLC is shown at the *bottom* of the lane.

Cross reactivity of LipB with noncognate C_8_-ACP substrates was also investigated. The enzyme assay comprised 5 μM *E. coli* LipB, an octanoate donor (C_8_-ACP, 75 μM), and an octanoate acceptor GcvH (25 μM). Three separate assays were performed using different octanoate-donors: (a) *E. coli* C_8_-ACP (UNP 0A6A8), (b) C_8_-*Lm*ACP (*L. major* ACP, UNP E9AD06), and (c) C_8_-*Pf*ACP (*P. falciparum* ACP, UNP Q7KWJ1). The product formed after the assay (C_8_-GcvH) in each case was qualitatively analyzed on a 12% SDS-PAGE gel (Fig. 2*A*). Lane 1 is pure apo-GcvH, lane 2: C_8_-*Ec*ACP, lane 3: C_8_-*Lm*ACP, lane 4: C_8_-*Pf*ACP (used as a reference/control). Notably, all three ACPs have a molecular weight of ∼8 kDa, but their migration pattern on SDS-PAGE suggests a much higher value. As shown in Figure 2*A*1, the molecular weight determined from its migration on the 12% SDS-PAGE gel is 18 kDa, consistent with several previous studies (30). ACP is an acidic protein that repels SDS, resulting in lower electrophoretic mobility on an SDS-PAGE gel. Lanes 6 to 8 display LipB assay carried out using C_8_-*Ec*ACP, C_8_-*Lm*ACP, and C_8_-*Pf*ACP, respectively, as donor substrates. Apo-GcvH to C_8_-GcvH conversion was discerned from the forward migration of the GcvH band on the SDS-PAGE gel. The conversion was quantitatively analyzed by running the samples on a C18 reversed phase HPLC column and integrating the area under the elution peak, shown in Figure 2, *B*–*D*. Approximately, 90% GcvH conversion was observed in presence of C_8_-ACP (*E. coli*) (Fig. 2*B*), 74% with C_8_-*Lm*ACP (*L. major* ACP) (Fig. 2*C*), and 85% when C_8_-*Pf*ACP (*P. falciparum* ACP) was used as an octanoate donor, illustrated in Figure 2*D*.

### LipB can octanylate GcvH using C_8_-CoA as a donor substrate

Next, we probed the importance of ACP scaffold in the substrate recognition by LipB. C_8_-CoA comprises a C_8_-chain and a phosphopantetheine arm, identical to the modifications present in C_8_-ACP. In addition, it contains an adenosine 3′5′-diphosphate group, not found in C_8_-ACP. To test whether LipB can use CoA analogs as a donor substrate, different lengths of acyl-CoA were used *viz.* C_4_-CoA, C_8_-CoA, C_10_-CoA, and C_12_-CoA, and the reaction progress was qualitatively monitored by following the modification of GcvH on an SDS-PAGE gel. Quantitatively, the reaction progress was followed by reverse phase HPLC chromatography. As shown in Figure 2*E*, LipB efficiently modified apo-GcvH using C_8_-CoA as acyl-chain donor (lane 5). No change in migration of the GcvH band was observed when C_4_-CoA, C_10_-CoA, or C_12_-CoA were used as donors, shown in lanes 4, 6, and 7, respectively in Figure 2*E*. MALDI-TOF mass spectrometry confirmed an increase in mass of GcvH by 128 Da after the enzyme assay, when C_8_-CoA was used as an acyl-chain donor. This change is mass is equivalent to the molecular mass of the C_8_-chain (127 Da, Fig. S5*C*). The expected molecular mass of apo-GcvH is 15,834 Da (GcvH subunit containing an N-terminal His tag “GSSHHHHSSGLVPRGSHM”). The terminal methionine of His tag was cleaved by *E. coli* MAP1, endogenous methionine aminopeptidase MAP (31). Fig. S5*A* shows the mass spectra for apo-GcvH (used as a reference) and Fig. S5, *B*–*E* mass spectra of GcvH after the LipB assay using C_4_-CoA, C_8_-CoA, C_10_-CoA, and C_12_-CoA as acyl-chain donors. No change in mass was observed in the assays performed with C_4_-CoA, C_10_-CoA, and C_12_-CoA (Fig. S5, *B*, *D* and *E*).

Mammalian LipT2, an octanoyltransferase is associated with vast number of neurological disorders. The ability of mice *Mm*LipT2 (*Mus musculus*, UNP Q9D009) to transfer octanoate from C_8_-CoA to *E. coli* GcvH was also investigated. As shown in Figure 2*F*, *Mm*LipT2 also successfully converted *E. coli* GcvH using C_8_-CoA as a donor substrate, which was confirmed from the forward migration of the GcvH band in the 12% SDS-PAGE gel. Molecular weight marker is shown in lane 1, *E. coli* apo-GcvH (used as reference) in lane 2, and *Mm*LipT2 assay products in lane 3.

**Figure 3:**
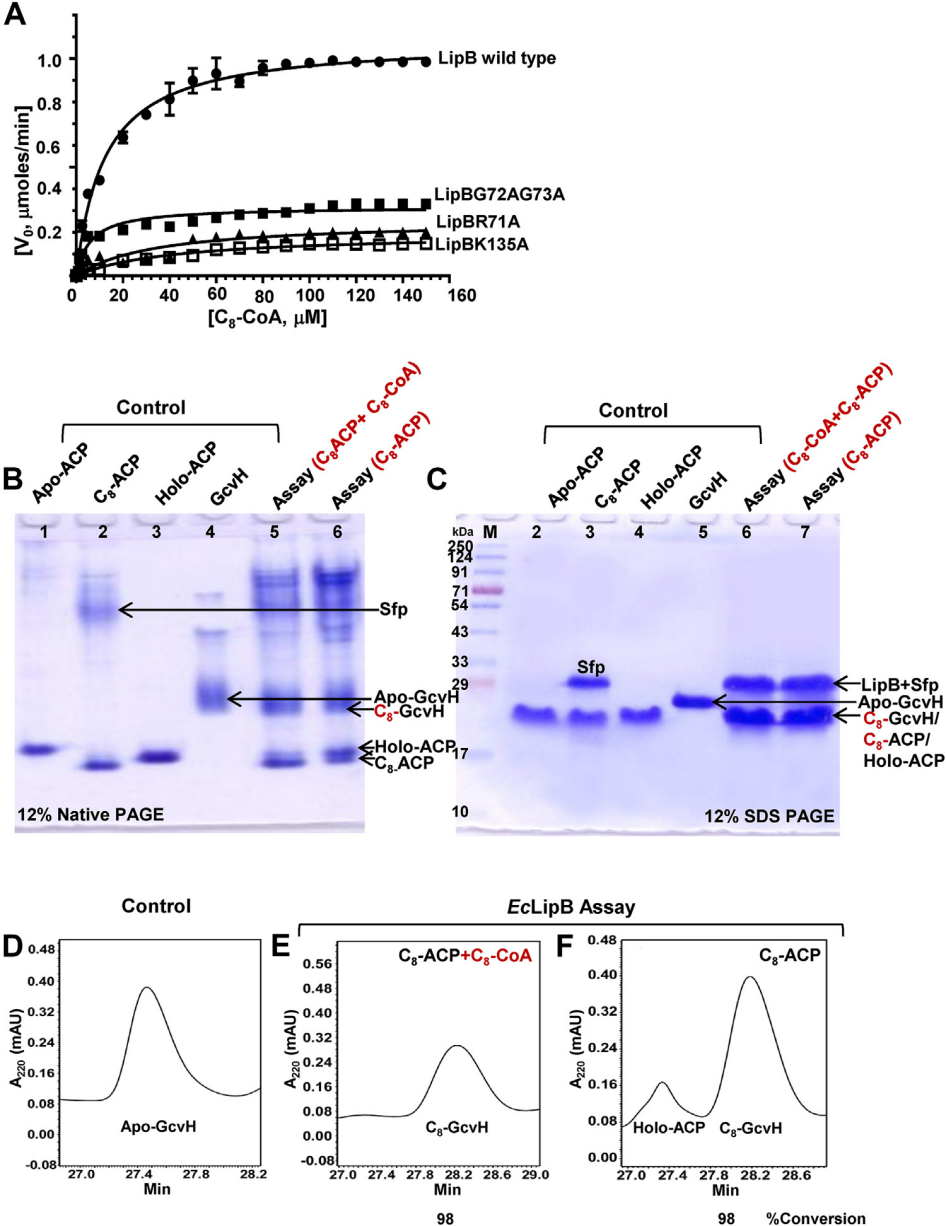
**LipB prefers C**_**8**_**-CoA over C**_**8**_**-ACP as a donor substrate.***A*, kinetic studies using -•- LipB, -▲- LipBR71A, -▪- LipBG72A/G73A, and -□-LipBK135A as octanoyl-transferases, C_8_-CoA as a donor substrate, and GcvH as an acceptor substrate. Reported values are the average of two independent measurements done on a Symmetry C18 reversed phase HPLC column. The figure was drawn using GraphPad prism version 7.0. *B*, a 12% native-PAGE gel displaying lane 1 to 4: apo-ACP, C_8_-ACP, holo-ACP, and apo-GcvH, respectively, used as controls. Lane 5 was loaded with LipB assay performed in presence of equimolar concentration of C_8_-ACP and C_8_-CoA used as donor substrates. Lane 6: assay done using C_8_-ACP alone as a donor substrate. *C*, the same samples were loaded on a 12% SDS-PAGE gel. Lane 1: Marker, lane 2 to 5: apo-ACP, C_8_-ACP, holo-ACP, and GcvH samples loaded as a reference. Lane 6: assay carried out using equimolar concentration of C_8_-CoA and C_8_-ACP, while lane 7 was loaded with the assay carried out in presence of C_8_-ACP alone. In both the assays, GcvH was used as an acceptor substrate for the bisubstrate reaction, and its conversion to C_8_-GcvH was followed by forward migration. C18-reversed phase chromatogram for (*D*) Apo-GcvH used as a reference, LipB assay in presence of (*E*) equimolar concentration of C_8_-ACP: C_8_-CoA, and (*F*) C_8_-ACP alone. Samples in Figure (*D*–*F*) were the same samples that were loaded in lanes 4 to 6 of the Native-PAGE and lanes 5 to 7 of SDS-PAGE gels in figures (*B*) and (*C*).

Kinetic measurements were also performed keeping LipB (5 μM) and GcvH (25 μM) concentration constant and varying the C_8_-CoA concentration (0–160 μM). The results were quantitatively analyzed on a C18-reversed phase column. Kinetic constants were obtained by performing hyperbolic Michaelis–Menten fits to the raw data, using GraphPad Prism version 7.0 (GraphPad Software), listed in Table 1. A *K*_m_ value of 12.2 ± 0.8 μM and a *V*_max_ of 1.1 ± 0.01 μmol min^−1,^ were obtained from the graph (*filled circles*), illustrated in Figure 3*A*. In a previous study, using C_8_-ACP as a substrate, a *K*_m_ of 10.2 ± 4.4 μM has been reported for LipB (17).

**Table 1:** Enzyme kinetic parameters for *E. coli* WT LipB and the mutants

Enzyme parameters	LipBWt	LipBG72A/G73A	LipBR71A	LipBK135A
*V*_max_ (μmol/min)	1.08 ± 0.01	0.32 ± 0.01	0.26 ± 0.02	0.20 ± 0.01
*K*_m_(μM)	12.24 ± 0.79	6.71 ± 0.98	34.39 ± 8.8	42.37 ± 4.58
*k*_cat_ (min^−1^)	0.22	0.06	0.05	0.04

The assay was performed using C_8_-CoA as octanoate donor and *E. coli* GcvH as octanoate acceptor.

### C_8_-CoA is preferred over C_8_-ACP as a LipB substrate

LipM, a LipB homolog from *B. subtilis*, prefers C_8_-ACP as a substrate and poorly utilizes C_8_-CoA (32). Does *E. coli* LipB behave in a similar manner? To address this question, competitive assays were performed using equimolar concentrations of C_8_-ACP and C_8_-CoA. The breakdown of C_8_-ACP to holo-ACP was followed on a 12% native-PAGE and conversion of apo-GcvH into its holo-form on a 12% SDS-PAGE. ACP intermediates were best visible on a native-PAGE, while apo-GcvH and holo-forms were more distinct on an SDS-PAGE gel. In Figure 3*B*, lanes 1, 2, 3, and 4 display the bands corresponding to apo-ACP, C_8_-ACP, Holo-ACP, and apo-GcvH (all four proteins used as reference), respectively, on a 12% native-PAGE. Lane 5 shows the assay performed in presence of equimolar concentration of C_8_-CoA and C_8_-ACP, and lane 6 displays bands for the assay carried out using C_8_-ACP alone as a donor substrate. It is noteworthy that in lane 5, a major proportion of C_8_-ACP remained unused after the assay, though GcvH displayed complete conversion. On the contrary, an intense band equivalent to holo-ACP appeared in lane 6, just above the C_8_-ACP band, in addition to the newly formed C_8_-GcvH band. Replicates of the same sample were also loaded on a 12% SDS-PAGE gel (Fig. 3*C*). Lanes 2 to 5 show bands for apo-ACP, C_8_-ACP, holo-ACP, and apo-GcvH, used as reference. Lane 6 corresponds to the assay carried out in presence of C_8_-CoA + C_8_-ACP, while lane 7 shows the bands for the assay with C_8_-ACP alone. Notably, both lanes 6 and 7 displayed complete conversion of apo-GcvH to C_8_-GcvH. However, based on native-PAGE (Fig. 3*B*), in lane 5, the intensity of the holo-ACP band was low compared to lane 6, suggesting less consumption of C_8_-ACP in presence of C_8_-CoA. C18-reversed phase chromatography confirmed the gel results. Figure 3*D* shows a chromatogram for apo-GcvH alone, while Figure 3*E* after the assay using C_8_-CoA + C_8_-ACP, displaying the peak for C_8_-GcvH formed as a product (corresponding to lane 5 of Fig. 3*B*). Figure 3*F* displays the assay carried out using C_8_-ACP as a donor substrate (lane 6 sample of Fig. 3*B*). A distinct peak for holo-ACP appeared due to the transfer of octanoyl-chain from C_8_-ACP to GcvH (Fig. 3*F*). This peak was not observed in the assay performed in a reaction mixture containing equimolar C_8_-ACP and C_8_-CoA concentration (Fig. 3*E*).

### LipB interacts with adenosine as well as phosphopantetheine arm of CoA

**Figure 4:**
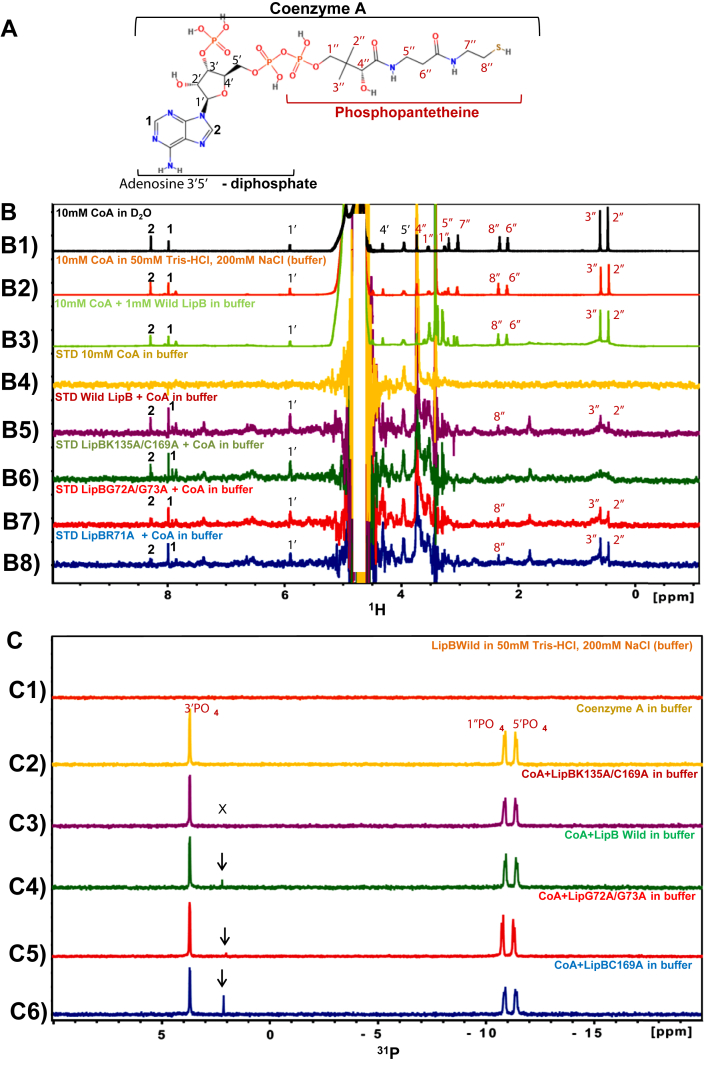
**LipB interacts with adenosine 3′5′-diphosphate and phosphopantetheine arm of CoA.***A*, the chemical structure of CoA is shown, displaying its two components phosphopantetheine (labeled *brown*) and ADP (labeled *black*). *B*1, ^1^H NMR spectra of CoA in D_2_O, (*B*2) ^1^H spectra of CoA in Tris–HCl, (*B*3) ^1^H spectra of CoA in Tris–HCl, in presence of 1 mM WT LipB. Saturation transfer difference (STD) spectra for (*B*4) CoA alone in buffer, (*B5*) CoA: wild LipB, (*B*6) CoA:LipBK135A/C169A, (*B*7) CoA:LipBG72A/G73A, and (*B*8) CoA:LipBR71A. *C*, ^31^P NMR spectra of (*C*1) WT LipB in Tris–HCl, (*C*2) CoA in Tris–HCl buffer, (*C*3) CoA: LipBK135A/C169A, (*C*4) CoA: WT LipB, (*C*5) CoA:LipBG72A/G73A, (*C*6) CoA:LipBC169A. The molar ratio of CoA:LipB was maintained at 10:1 in all STD experiments and 8:1 in the ^31^P NMR experiments.

C_8_-CoA contains an adenosine-3′,5′-diphosphate group, in addition to the C_8_-chain, and the phosphopantetheine moiety. From the biochemical assays, it was unclear whether CoA component of C_8_-CoA also interacts with LipB. Therefore, LipB:CoA binding studies were carried out using NMR at 283 K. The chemical structure of CoA is shown in Figure 4*A*, with all its nonexchangeable proton resonances labeled. Amide protons of CoA could not be observed in 90% H_2_O sample due to hydrogen–deuterium exchange. Proton chemical shifts of CoA were assigned based on the BMRB ID bmse000271. Figure 4*B*1 illustrates the ^1^H NMR spectra of CoA in D_2_O (used as a reference). The sample displays three peaks in the 6.0 to 9.0 ppm range, corresponding to 1, 2, and 1′ resonances of the adenosine component of CoA (atom numbering shown in Fig. 4*A*). In the 0 to 5 ppm range, 4′ and 5′ peaks from the adenosine component (colored *black*) and 1", 2", 3", 4", 5", 6", 7", and 8" peaks arising from the phosphopantetheine arm were also observed. Figure 4*B*2 displays the NMR spectra for CoA in buffer (50 mM Tris–HCl, 200 mM NaCl, pH 7.8). Figure 4*B*3 shows the ^1^H NMR spectra for CoA in the same Tris–HCl buffer pH 7.8, in presence of 1 mM WT LipB (for reference). Figure 4*B*4 illustrates the saturation transfer difference (STD) spectra for CoA in buffer to ensure no intramolecular NOE transfer occurs. Figure 4, *B*5–*B*8 displays the STD spectra for CoA in the presence of WT LipB, LipBK135A/C169A (catalytically inactive double mutant), LipBG72A/G73A, and LipBR71A, respectively. All these STD experiments were acquired using a CoA:LipB molar ratio of 10:1. NOEs corresponding to position 1, 2, and 1′ were observed in Figure 4, *B*5–*B*8, suggesting binding of the adenosine ring. Similarly, in the 0 to 5 ppm range, peaks 2", 3", and 8" corresponding to the phosphopantetheine arm displayed NOEs in the STD spectra in presence of WT and the mutant LipB. The on resonance (*red*), off resonance (*blue*),and STD spectra (*green*) for the binding of LipBK135A/C169A with CoA in 50 mM Tris, 200 mM NaCl, pH 7.8 is illustrated in Fig. S6*B*. Fig. S6*A* shows the chemical structure of CoA.

^31^P NMR studies were also performed to understand the interaction of LipB with the phosphates of CoA. Figure 4*C*1 shows the ^31^P NMR spectra for 1 mM LipB in 50 mM Tris, 200 mM NaCl, pH 7.8 at 298 K. No peaks were observed, suggesting protein as such does not contain any phosphate group. Figure 4*C*2 displays the ^31^P spectra for CoA in 50 mM Tris, 200 mM NaCl, pH 7.8. Three prominent peaks were visible, corresponding to 3′-phosphate, 1"-phosphate, and 5′- phosphate. Figure 4, *C*3–*C*6 show the ^31^P spectra for CoA in presence of LipBK135A/C169A, WT, LipBG72A/G73A, and LipBC169A, respectively. The peak corresponding to 3′-phosphate displayed an upfield change in chemical shift upon addition of WT LipB (Fig. 4*C*4), LipBG72A/G73A (Fig. 4*C*5), and LipBC169A (Fig. 4*C*6). The new peak arising as a result of this interaction is indicated by a downward pointing arrow in the figures. No change in peak position was observed in LipBK135A/C169A sample (Fig. 4*C*3), suggesting interaction of Lys135 with 3′-phosphate of CoA, that is lost in the double mutant. A CoA:LipB ratio of 8:1 was used for these experiments.

### Are adenine- and phosphate-binding sites also conserved in LipB?

^15^N-labeled *E. coli* LipBK135A/C169A (catalytically inactive double mutant) was titrated with C_8_-CoA, and the titration was followed by NMR. Several amides in the ^15^N TROSY-HSQC experiment displayed significant chemical shift change (Fig. S7). In the figure, LipBK135A/C169A 1:0 M ratio (*red*), 1:0.2 (*cyan*), 1:0.4 (*yellow*), 1:0.8 (*green*), 1:1 (*magenta*), and 1:2 (*orange*) are shown as multiple overlaid spectra. The direction of chemical shift change is shown by a *black arrow*. Most of the peaks displaying perturbations appeared partially broadened due to chemical exchange. Unfortunately, owing to the low stability of the enzyme at high concentrations (necessary for backbone assignments), we were unable to assign the backbone amides of *E. coli* LipB. The pattern of amide perturbations though confirmed that *E. coli* LipB binds C_8_-CoA.

Notably, the lipoate scavenging enzyme LplA has adenine- and phosphate-binding sites that favor lipoic acid and ATP binding. We asked whether adenine and phosphate binding sites are also conserved in LipB, given the evolutionary relationship of the two proteins. Thus, *M. tuberculosis Mt*LipB structure (PDB 1W66) was superimposed on the catalytic domain of *E. coli* LplA complexed with C_8_-AMP (LplA, PDB 3A7A), using the matchmaker option of Chimera, shown in Fig. S8*A* (33). In most cases, atoms N2, N6, C2, and C8 of adenine, present at the Watson–Crick edge interact with the backbone and side chain atoms of adenine-binding proteins (34). In the *E. coli* LplA structure (PDB 3A7A), N1 and N6 of adenine form a hydrogen bond with the side chain of Asn 83 OD1, Thr 151, and the backbone O of Phe 78. Also, N7 forms a hydrogen bond with the backbone N of Phe 78. In *M. tuberculosis* LipB (*Mt*LipB, 1W66), remarkably similar residues are present at some of these sites, Gln 87 and Trp 82 (instead of Asn 83 and Phe 78 in LplA). In *E. coli* LipB, Gln 82 and Tyr 77 are present, while *Mm*LipT2 possesses Gln 96 and Phe 91 at the same position in the sequence. The oxygen atoms of AMP (O4 and O5) interact with the ε amino group of Lys 133 in *Ec*LplA. The equivalent residue is Lys 142 in *Mt*LipB and Lys 135 in *E. coli* LipB. Also, in the LplA structure (PDB 3A7A), lipoyl moiety interacts with numerous hydrophobic/aromatic side chains, *viz.* Trp 37, Val 44, Arg 70, Val77, His79, and His149. In *Mt*LipB, these positions are occupied by Leu 47, Thr 54, Arg 76, Thr 81, His 83, and His 157 in the structure (Fig. S8*B*). In *E. coli* LipB, Val 41, Thr 48, Arg 71, Thr 76, His 78, and His 150 are present at the same position in the sequence, shown in Fig. S8*B*.

The lipoate-binding loop ^69^RRXXGGG^75^ of LplA (PDB 3A7A) is also somewhat conserved in *Mt*LipB and has a very similar conformation, shown in Fig. S8, *C* and *D* (35). *Mt*LipB backbone and side chain are colored *cyan*, while LplA molecule is colored *brown*. Arg 70 side chain and Gly 73 backbone of LplA (PDB 3A7A) structurally overlaps with Arg 76 and Gly 78 of *M. tuberculosis* LipB (PDB 1W66). Arg 71 and Gly 72 are the equivalent residues in *E. coli* LipB. Interestingly, LplA Arg 70 side chain forms unique side chain to backbone hydrogen bonds with the carbonyls of Gly73, Gly75, and side chain of Ser72 in the LplA structure (Fig. S8*D*, colored *brown*). These interactions have been speculated to stabilize the lipoate-binding loop of LplA. In *Mt*LipB as well, arginine 76 side chain forms identical hydrogen bonds with the backbone carbonyls of Gly 77 and Lys 79, colored *cyan* in Fig. S8*D*. The structural similarities between LipB and LplA hint at the possibility of conservation of adenine- and phosphate-binding sites in LipB, like LplA.

### 'RGG' loop is necessary for full LipB activity

**Figure 5:**
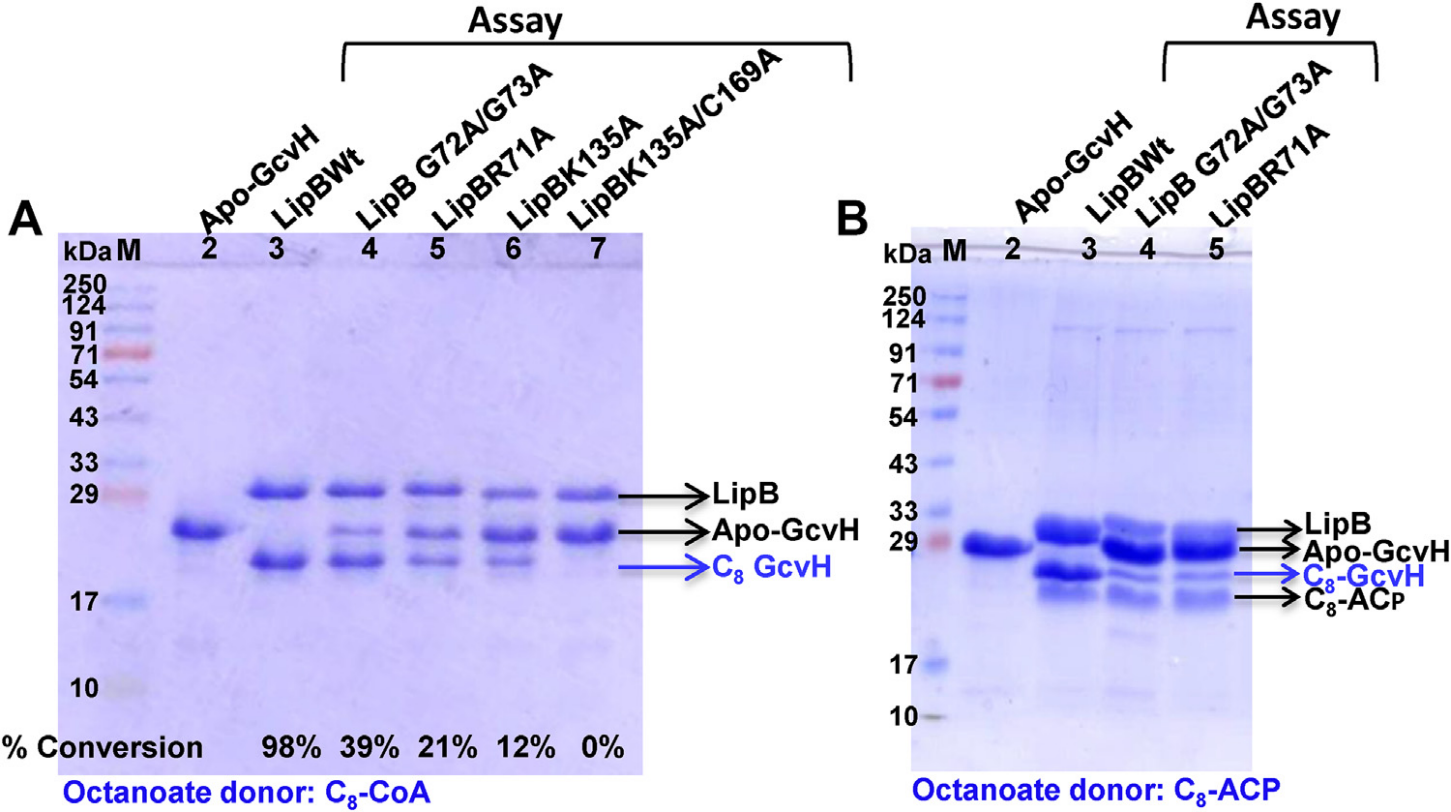
**RGG loop is necessary for full LipB activity.***A*, a 12% SDS-PAGE gel displaying molecular weight marker in lane 1, lane 2: apo-GcvH alone, lanes 3 to 7: assay carried out in presence of WT LipB, LipBG72A/G73A, LipBR71A, LipBK135A, and LipBK135A/C169A, respectively, using C_8_-CoA as an octanoate donor. Percent conversion of GcvH after the assay is shown at the *bottom* of each lane. *B*, a 12% SDS-PAGE gel, displaying lane 1: marker, lane 2: apo-GcvH, lane 3 to 5: assay carried out with WT LipB, LipBG72A/G73A, and LipBR71A, using C_8_-ACP as a donor substrate.

The importance of RGG loop in LipB function was investigated by generating LipBR71A and LipBG72A/G73A mutants and performing enzyme assays using C_8_-CoA as octanoyl donor and GcvH as acceptor substrate. Figure 5*A* shows a 12% SDS-PAGE gel, displaying molecular weight marker in lane 1, purified apo-*Ec*GcvH in lane 2 (used as a reference), and assays with WT LipB, LipBG72A/G73A, LipBR71A, LipBK135A, and LipBK135A/C169A, respectively, in lanes 3 to 7. Apo-GcvH to C_8_-GcvH conversion was ascertained from the forward migration of the GcvH band in the gel. Compared to WT LipB, GcvH conversion was remarkably reduced in lanes 4, 5, 6, and 7, suggesting loss of activity of the mutants we tested. LipBG72A/G73A displayed 39%, while LipBR71A displayed 21% activity compared to WT LipB, the values determined using C18 HPLC reversed-phase chromatography.

Similar LipB assays were also performed using C_8_-ACP as an octanoate donor and GcvH as acceptor. A marked reduction in enzyme activity was observed in LipBG72A/G73A and LipBR71A mutants compared to WT LipB (Fig. 5*B*). In the 12% SDS-PAGE gel, lane 1 is molecular weight marker, lane 2 apo-GcvH (used as a reference), and lanes 3 to 5 assay performed with WT LipB, LipBG72/G73A and LipBR71A mutants, respectively. C_8_-GcvH band was most intense in lane 3 and relatively faint in lanes 4 and 5.

Kinetic measurements were performed on LipBR71A (*filled triangles*) and LipBG72A/G73A mutants (*filled squares*), using C_8_-CoA as an octanoate donor, shown in Figure 3*A*. A *K*_m_ value of 6.7 ± 0.9 μM (a twofold decrease) and a *V*_max_ of 0.32 ± 0.01 μmol min^−1^ (approximate threefold decrease compared to WT) was observed for the LipBG72A/G73A mutant. LipBR71A mutant displayed a *K*_m_ value of 34.4 ± 8.8 μM (approximately threefold increase) and a *V*_max_ of 0.26 ± 0.02 μmol min^−1^ (fourfold decrease, Fig. 3*A*). In comparison, LipBK135A mutant displayed a *K*_m_ of 42.4 ± 4.6 μM (3.5-fold change) and a *V*_max_ of 0.2 ± 0.01 μmol min^−1^ (Table 1).

STD experiments were also performed to monitor the binding of CoA with LipBG72/G73A and LipBR71A mutants. Figure 4, *B*7 and *B*8 display 1D STD spectra for CoA:LipBG72A/G73A and CoA:LipBR71A interaction. NOEs corresponding to the resonances of adenosine and phosphopantetheine were visible in both the spectra, suggesting binding of CoA with the mutants. Figure 4*C*5 shows the ^31^P NMR spectra for the interaction of CoA with LipBG72A/G73A mutant. The 3′-phosphate peak displayed a chemical shift change similar to WT protein, suggesting phosphate binding.

### Molecular dynamics simulations suggest RGG sequence imparts conformational flexibility to the LipB active site loops

**Figure 6:**
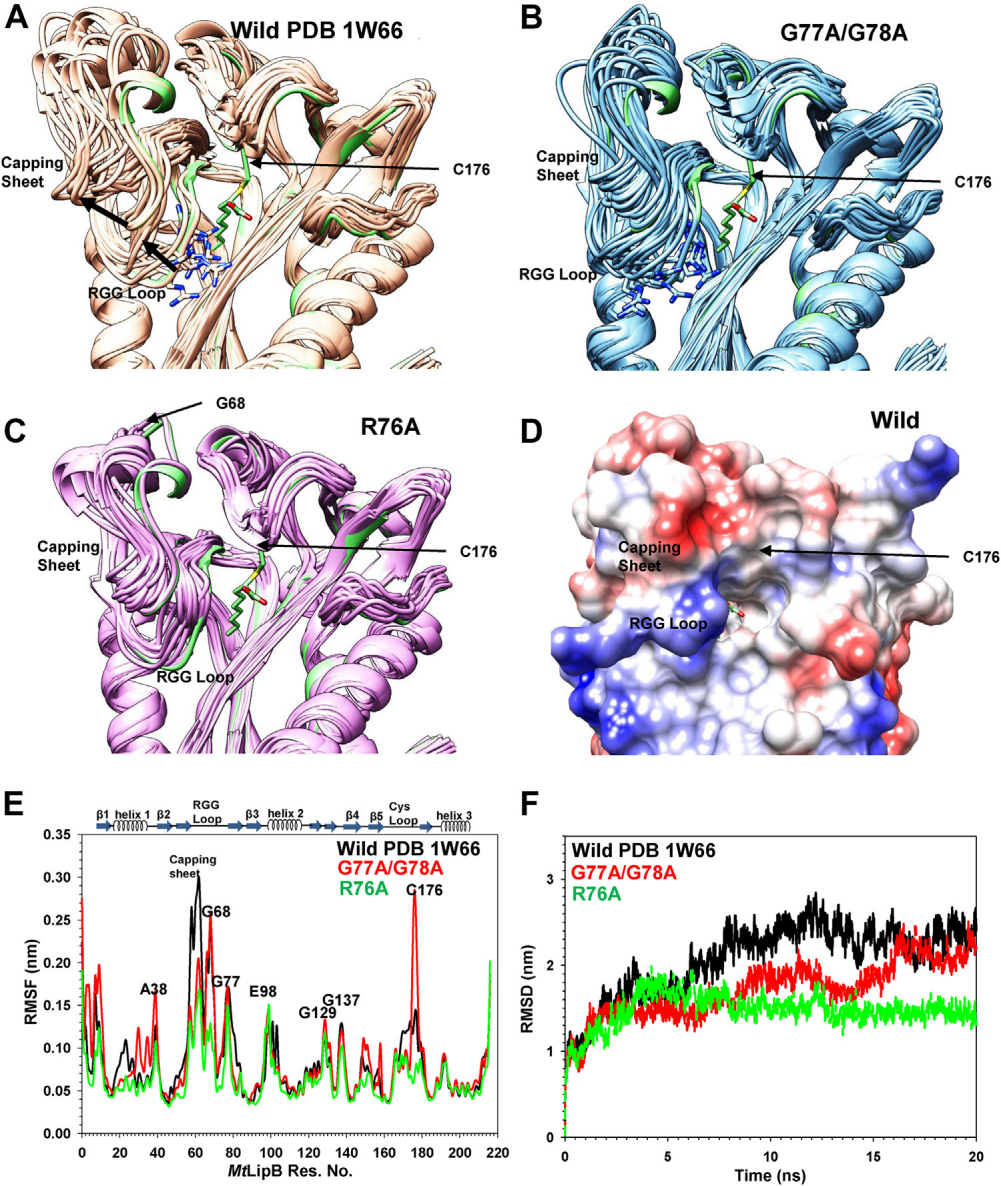
**Molecular dynamics simulations of WT LipB and the mutants.** Superposition of the 21 structures obtained at the end of GROMACS 20 ns simulations for (*A*) WT LipB, (*B*) LipBG77A/G78A, and (*C*) LipBR76A. *D*, the surface of wild type LipB is shown, displaying the active site cavity. *E*, root mean square fluctuation (CαRMSF) as a function of residue number, and (*F*) root-mean-square deviation (CαRMSD) for WT LipB (represented by *black lines*), LipBG77A/G78A (*red line*) and LipBR76A (*green line*) during the simulations.

The lipoate-binding ^69^RRXXGGG^75^ loop of LplA experiences large scale conformational changes that are necessary for binding its substrate (35). Therefore, molecular dynamics (MD) simulations were performed on WT LipB and its RGG mutants using GROningen Machine for Chemical Simulations (Gromacs 2021.5; University of Groningen; Royal Institute of Technology; Uppsala University) to understand the role of the loop in backbone dynamics (36). PDB 1W66 was edited using the swapaa command of Chimera in order to generate LipBR76A and LipBG77A/G78A mutant structures, and the resulting PDB files were saved and stored for GROMACS simulations. Figure 6, *A*–*C* display the conformation of the capping sheet, 'RGG' loop, and cysteine containing loop in the 21 models generated using 20 ns MD simulations on PDB 1W66 (WT), *Mt*LipBG77A/G78A, and *Mt*LipBR76A mutants. In both WT and *Mt*LipBG77A/G78A simulations, Arg 76 side chain was observed to flip out of the active site cavity as a function of time (Fig. 6, *A* and *B*). During the course of simulations, capping sheet and the RGG loop were observed moving upward, away from the active site in WT LipB (Fig. 6*A*), widening the mouth of the cavity. The starting structure used for simulations is shown as a *green* ribbon. The direction of capping sheet and loop movement is shown by *black* arrows. In *Mt*LipBG77A/G78A mutant, the capping sheet displayed restricted movement, but the loop containing C176 displayed reasonable change. In *Mt*LipBR76A mutant, motions were restricted in both Cys176 containing loop as well as the capping sheet, with all the loops in the structures converged (Fig. 6*C*). Figure 6*D* shows the molecular surface of WT LipB, displaying the active site cavity tunnel, and the RGG loop masking it. We speculate that the movement of the RGG loop away would widen the mouth of the active site. Figure 6*E* displays root mean square fluctuation (RMSF) values as a function of residue number for WT *Mt*LipB (PDB 1W66, *black lines*), *Mt*LipBR76A (*green lines*), and *Mt*LipBG77A/G78A mutant (*red lines*) simulations. In the WT protein, there were large magnitude RMSF fluctuations in the capping sheet and the RGG loop. The motions in the capping sheet were subdued in *Mt*LipBG77A/G78A and *Mt*LipBR76A mutants. Figure 6*F* shows the rmsd values for the three proteins as a function of time. All the structures converged within 20 ns simulation time, though *Mt*LipBR76A converged relatively faster and at a much lower rmsd value. Overall, our results highlight the possible role of RGG loop in regulating the movement of the capping sheet and maintaining the conformation of the Cys176 loop, thus, making active site accessible to the substrate and releasing the product.

## Discussion

In prokaryotes, two different pathways for lipoic acid modification coexist: (a) an endogenous lipoic acid synthesis pathway, catalyzed by the consecutive action of LipB and lipoyl synthase (LipA) or (b) a salvage pathway carried out by LplA. Yeast and higher eukaryotes lack the scavenging pathway and derive lipoic acid exclusively from the biosynthetic pathway by the sequential action of LipT2, LIAS, and LipT1 (5). The three enzymes work in concert, performing nonredundant functions of the mitochondria. Genetic mutation of any one of these or the upstream type II fatty acid synthesis enzyme causes metabolic dysfunction (37, 38). The disorders cannot be treated by supplementation with exogenous lipoic acid, as all three enzymes cannot uptake free lipoic acid and the cofactor has to be synthesized on-site. Thus, there is urgent need to understand lipoic acid synthesis at the molecular level (11, 21, 22).

LipT2, LIAS, and LipT1 are mammalian homologs of *E. coli* LipB, LipA, and LplA. LipB (the biosynthetic enzyme) and the salvage enzyme LplA differ from each other in several respects: (a) C_8_-ACP, an intermediate of the fatty acid synthesis pathway is the only known substrate of LipB, while LplA is a lipoic acid/ATP-dependent enzyme; (b) a cysteine-lysine dyad serve as catalytic residues of LipB, whereas a lysine performs lipoate adenylation as well as transfer reactions of LplA (35); (c) reaction proceeds *via* different intermediates in the two enzymes; LplA forms a noncovalent lipoyl-5′-AMP intermediate from lipoic acid and ATP, while a C_8_-LipB covalent adduct is formed using mitochondrial fatty acid synthase intermediate (C_8_-ACP). Despite these dissimilarities, unique parallels appear to exist between the two enzymes with regard to substrate recognition, as revealed by our studies. STD experiments and ^31^P NMR studies suggest binding of adenosine and phosphopantetheine moiety of CoA to LipB, similar to LplA (39). Stronger affinity of holo-ACP for LipB compared to apo-ACP and perturbations in one of the NH resonances of its phosphopantetheine moiety upon LipB titration support the conservation of a phosphate-binding site in LipB, like LplA. Structural overlay of *Mt*LipB (1W66) on the catalytic domain of *E. coli* LplA (3A7A) also hints toward conservation of residues in LipB at sites that interact with adenine and phosphate groups of AMP in LplA structures (PDB 3A7A, 2ART, 2ARU). Interestingly, the two sites are conserved in the sequence of lipoate transferase 2 (LipT2, UNP A6NK58) as well, and the latter enzyme also converts GcvH using C_8_-CoA, divulging evolutionary relationships (32).

The salvage enzyme LplA interacts with lipoic acid *via* a well-defined lipoate-binding loop. An ^76^RGG^78^ loop present in the active site of (*M. tuberculosis*) *Mt*LipB (1W66) has sequence and conformation fairly similar to the lipoate-binding loop ^69^RRSSGGGAV^77^ of LplA. In the LplA structure (PDB 3A7A), Arg 69 forms unique side chain to backbone hydrogen bonds with the glycine and Ala of the loop, in a manner similar to Arg 76 of LipB (*M. tuberculosis*). This lipoate-binding loop is crucial for lipoylation in LplA and experiences large scale conformational changes upon lipoic acid binding (35). MD simulations of *M. tuberculosis* LipB (PDB 1W66) suggest similar motions in the capping sheet and the RGG loop that are quenched upon mutation of Arg 76 in the sequence to alanine. Conversely, mutation of the conserved glycines in the RGG sequence to Ala increased the overall dynamics of the Cys176 (catalytic cysteine) loop, while the capping sheet displayed reduced motions, inducing major changes in the conformation of the active site. Interestingly, 'RGG' sequences are frequently found in RRM domains and are known to bind phosphates of nucleotides/quadruplexes (40). Electrostatic attraction/hydrogen bonds between positively charged guanidinium of arginine and the negatively charged phosphates contribute to this interaction (41, 42). Interaction between phosphates and phosphotransacetylase enzyme from *Methanosarcina thermophila* has also been attributed to arginines (43). We surmise that the 'RGG' loop of LipB regulates the active site opening of LipB and may be transiently involved in interaction with the phosphate group of its donor substrate, owing to the side chain motions of Arg 76 during simulations.

Taken together, the present study highlights the importance of ACP modifications in substrate recognition by LipB. Apo-ACP weakly binds LipB, while the presence of a phosphopantetheine moiety (in holo-ACP) allows ACP to bind LipB with several-fold higher affinity. Addition of a C_8_-chain further increases the binding affinity. These are new observations, shedding light on the unique mechanism of substrate recognition by LipB. We show that the phosphopantetheine moiety, a component of the substrate C_8_-ACP not just serves as a swinging arm to transfer acyl chain to the active site cavity of FAS enzymes but also plays a crucial role in substrate recognition by LipB. In the absence of the phosphopantetheine group, binding of ACP and LipB is remarkably weak. The present work complements a previous study on LipB that emphasizes the importance of surface complementarity between C_8_-ACP and LipB in substrate recognition by LipB. Based on that study, LipB can differentiate between different acyl intermediates allosterically, without even flipping the acyl chain (28, 29).

Above all, in the present work, we have identified a new substrate analog of LipB, that is C_8_-CoA, which can also serve as a substrate for LipT2. This information might be of assistance while treating patients with human mitochondrial fatty acid synthase mutations; genes encoding mitochondrial *trans*-2-enoyl-coenzyme A-reductase (MECR) (21, 44), malonyl-CoA synthetase (ACSF3), malonyl CoA-acyl carrier protein transacylase (MCAT), etc. characterized by neurological manifestations due to the loss of lipoylation (21). The symptoms overlap with CoPAN and PKAN disorders (defects in CoA biosynthesis), as all these conditions have the same consequence, that is, reduction of lipoylation (25). Two candidates have been proposed as a therapy so far, lipoic acid and octanoic acid. Studies with radioactive lipoic acid supplements show that exogenous lipoic acid enters the blood stream and tissues of mammals but is rapidly degraded by β-oxidation with no noticeable lipoic acid modification (5). Likewise, in LipT2, LIAS, or LipT1 KOs, lipoic acid supplements do not provide any respite (5). We surmise that octanoic acid analogs might serve as a better substitute for C_8_-ACP, owing to their ability to pass through the mitochondrial membrane, convert into octanoyl-CoA inside the mitochondria, and act as a donor substrate for LipT2. Support for this proposition also comes from studies on individuals with medium chain acyl-CoA dehydrogenase deficiency, a fatty acid oxidation defect characterized by elevated C_8_-carnitine concentration, and lack of acyl-CoAs >C_10_-CoA. These individuals display increased lipoylated PDC levels compared to healthy controls, associating medium chain CoA to lipoic acid synthesis (45). Another recent study on yeast with deleted 3-hydroxyacyl thioester dehydrogenase, an important enzyme of mitochondrial FAS (Δhtd2) but harboring a mitochondrially mislocalized fatty acyl-CoA ligase (Fam1-1 suppressor allele) was able to rescue the cells by growth on C_8_-supplemented media. FAM1-1 has long back been shown to activate fatty acids by attachment to CoA or ACP (46). Authors attributed the suppression of respiratory growth defects in cells lacking mtFAS enzymes by Fam1-1 to a LipB-dependent mechanism, as the cell growth could not be achieved in the absence of lipoylating enzymes or C_8_-supplements (47). Our *in vitro* studies validate their findings, offering proof of concept that LipT2 can indeed efficiently use C_8_-CoA as a substrate, besides C_8_-ACP. Further studies are warranted, to test the efficacy of octanoate in rescuing FAS-deficient mutants.

## Experimental procedures

### Cloning and mutagenesis

Genes encoding ACP, LipB, and GcvH were PCR amplified from the *E. coli* genome and cloned in a pET28a expression vector between NdeI and HindIII restriction sites. The corresponding plasmids have been named as follows: pET28a(+)-*Ec*ACP, pET28a(+)-*Ec*LipB, pET28a(+)-*Ec*GcvH. Mice LipT2 gene (synthesized after codon optimization) was also cloned similarly (pET28a(+)-*Mm*LipT2). Mutagenesis studies were performed using site-directed mutagenesis approach using Pfu turbo as the polymerase and pET28a(+)-*Ec*LipB as a template. The PCR product was subjected to DpnI digestion overnight at 25 °C, in order to digest the methylated circular DNA. Approximately 15 μl of this product was transformed in XL-I blue competent cells. Colonies obtained were inoculated in 5 ml LB media, followed by overnight culture, pelleting, and finally plasmid isolation. For the double mutant, LipB plasmid containing lysine mutation was used as a template. All the mutations (*Ec*LipBK135A, *Ec*LipBC169A, *Ec*LipBK135A/C169A, *Ec*LipBR76A, and *Ec*LipBG72A/G73A) were confirmed by DNA sequencing.

### Protein expression

pET28a(+)-*Ec*ACP and pET28a(+)-*Ec*LipB plasmids were transformed separately into *E. coli* BL21(DE3) cells and plated on LB agar supplemented with 50 μg/ml of kanamycin. One colony of each was inoculated in 5 ml of LB media supplemented with kanamycin (50 μg/ml). The primary culture was incubated at 37 °C for 14 to 16 h, accompanied with shaking at 200 rpm. For large scale expression, 1 l LB medium (secondary culture supplemented with 50 μg/ml kanamycin) was inoculated with 1% of the overnight grown primary culture. The secondary culture was kept at 37 °C with shaking until its *A*_600_ reached a value of 0.6 to 0.8. For ACP and LipB expression, secondary culture was induced with 0.5 mM IPTG and incubated at 25 °C for 14 to 16 h, with shaking at 200 rpm. Post induction, cells were harvested by centrifugation at 4000 rpm at 4 °C for 30 min, and the pellet was used for protein purification.

### ^15^N ^13^C-labeled protein expression

The protocol for transformation and primary culture were exactly the same as described for the unlabeled protein. However, for the secondary culture, 500 ml M9 media was prepared by dissolving Na_2_HPO_4_ (3.4 g), KH_2_PO_4_ (1.5 g), NaCl (0.25 g), and NH_4_Cl (0.5 g) in distilled water. The media was autoclaved and cooled. Prior to culture inoculation, 1 g of unlabeled/labeled glucose (40%), 1 ml of 1 M MgSO_4_, 10 μl of 1 M CaCl_2_, 500 μl of 0.5% thiamine HCl, 500 μl of 50 mg/ml kanamycin, and 5 ml of vitamins (BME stock, Sigma) were added. To the 500 ml culture media, 4 ml of primary overnight grown culture was added. The secondary culture was kept at 37 °C with shaking until its *A*_600_ reached a value of 0.6 to 0.8. For ACP, culture was incubated at 25 °C for 14 to 16 h, with shaking at 200 rpm. Post induction, cells were harvested at 4000 rpm at 4 °C for 30 min, and the pellet was used for protein purification.

### Purification of ACP

Pellets were suspended in 30 ml of 20 mM sodium phosphate buffer (pH 7.8) and 500 mM NaCl. 1× EDTA free protease inhibitor cocktail (Sigma) was added to the suspended pellet and subjected to sonication (5 s ON, 5 s OFF, and 30% amplitude). As *Ec*ACP is expressed in both apo- and holo-forms, an additional phosphodiesterase activation step was introduced. The sonicated cells were incubated with 10 mM MgCl_2_ and 2 mM MnCl_2_ at 35 °C, 120 rpm for 2 h. Both, Mg^2+^ and Mn^2+^ ions were used to activate the natural *E. coli* phosphodiesterase, thereby converting holo-ACP to apo-ACP, and enriching the apo-ACP concentration in the lysate (48). Sonicated cells were centrifuged at 10,000 rpm, and the supernatant was passed through a Ni^2+^-NTA (Sigma) column, equilibrated with 20 mM sodium phosphate buffer, pH 7.8, containing 500 mM NaCl (washing buffer). The protein-bound resin was washed with 100 ml of washing buffer, followed by 100 ml of 20 mM sodium phosphate, pH 6.0, 500 mM NaCl wash (elution buffer) to remove the nonspecifically bound proteins. Next, the protein was eluted with imidazole in elution buffer and concentrated by 3 kDa cutoff centricons and imidazole removed. For the NMR samples, N-terminal His tag of ACP was cleaved by incubating with immobilized thrombin beads at 25 °C overnight while shaking at 120 rpm, post imidazole removal. In the subsequent step, salt concentration was decreased to 0 mM by washing the protein with 20 mM sodium phosphate buffer pH 6.0.

Since both apo- and holo-forms of the protein were present in the purified ACP fraction, the sample was subjected to ion-exchange chromatography using Resource Q column and eluted with a linear salt gradient of 0 to 500 mM NaCl in 20 mM sodium phosphate buffer, pH 6.0, 2 mM DTT. Separated apo- and holo-ACP were passed through size-exclusion columns to attain maximum purity, using 20 mM sodium phosphate buffer, 100 mM NaCl, pH 6.0.

### Purification of LipB

Cell pellets were suspended in 50 mM Tris–HCl, pH 8.0, 10% glycerol, and 300 mM NaCl (resuspension buffer). Suspended cells were subjected to sonication (5 s ON, 5 s OFF, and 30% amplitude). After sonication, cells were centrifuged at 10,000 rpm, and the supernatant was passed through Ni^2+^-NTA column, pre-equilibrated with resuspension buffer. The column was washed with the same buffer with 5 mM imidazole to eliminate nonspecific proteins. The protein of interest was eluted with 50 to 500 mM imidazole in resuspension buffer. The eluted fractions containing LipB were identified on a 12% SDS-PAGE gel (49) and concentrated using a 10 kDa centricon. The concentrated protein was passed through a Superdex 75 FPLC column (size-exclusion chromatography) equilibrated with 50 mM Tris–HCl, pH 7.8, 200 mM NaCl, and 10% glycerol. Pure fractions based on SDS-PAGE were pooled, concentrated, and stored in 50 mM Tris–HCl, pH 7.8, 200 mM NaCl, and 50% glycerol at −20 °C. For NMR titration studies, glycerol was removed completely prior to the experiments.

Most of the proteins were purified within 1 day starting from pellet, specially *Ec*LipB WT enzyme as well as the mutants. In case of *Ec*ACP, purification took nearly 2 days at 4 °C.

Apo-ACP was converted to holo-ACP and C_8_-ACP *in vitro*, using *B. subtilis* phosphopantetheinyl transferase (Sfp), using a modified protocol by Lambalot and Walsh (50). Sfp was expressed and purified by ion-exchange chromatography, followed by size-exclusion chromatography. CoA was purchased from Sigma (catalog no.: #C3144, ≥85% purity). C_8_-CoA (Sigma catalog no.: #O6877, purity ≥ 95%).

### NMR data acquisition

NMR samples comprised uniformly labeled [^1^H,^15^N,^13^C] protein, in 50 mM Tris–HCl, 200 mM NaCl, pH 8.0, 90% H_2_O, and 10% D_2_O, 0.5% sodium azide. All the spectra were acquired on a Bruker Avance III 700 MHz NMR spectrometer, installed at the National Institute of Immunology, equipped with triple resonance TXI probe. Triple resonance NMR experiments, *viz.* HNCA, HNCACB, CBCAcoNH, etc. were used to assign apo-, holo-, and C_8_-ACP. ^1^H^15^N TROSY-HSQC spectra were acquired using 1024 data points in the *t*_2_ dimension and 256 to 512 data points in the *t*_1_ dimension. NMR data were processed on a workstation running Red Hat Enterprize Linux 5.0, using NMRDraw/NMRPipe, and analyzed using Sparky (51). Experiments were performed at 298 K or otherwise as specified. ^15^N^13^C spectra were indirectly referenced using a chemical shift standard, sodium 2,2-dimethyl-2-silapentane-5-sulfonate (52).

### STD experiments

Saturation transfer difference experiments were acquired at 283 K using Bruker parameter sets for STDDIFFESGP.3, a pseudo-2D experiment, and analyzed using Topspin 2.1pl3 (Bruker's standard NMR software). A train of 50 ms Gaussian-shaped radio frequency pulses, attenuation level 55 dB, separated by intervals of 2 ms were used. Excitation sculpting was employed to suppress the water signal. The frequency for selective radiation for on resonance was placed at −3 ppm (for upfield shifted methyl protons) and off resonance at 35.5 ppm, respectively. A relaxation delay of 5 s was used. The acquisition time was 0.73 s. Subtracting on from off signal gave a positive STD signal from the bound CoA in the difference spectra. The ligand peaks observed in the 1D STD experiment were assigned based on ^1^H NMR spectra of CoA.

### ^31^P NMR measurements

^31^P NMR measurements were performed on a 5-mm broad band inverse probe, at 298 K, using Bruker ZG30 parameter set. A spectral width of 400 ppm, 65,536 data points, 2.0 s recycle delay, 14,336 scans, and 0.288 s acquisition time were used. A 30° flip angle was used in the experiment, with no proton decoupling. All the spectra were processed with a line broadening of 1.0 Hz, and phase correction using Topspin 2.1pl3 software.

### NMR data analysis

Chemical shift perturbations have been reported as weighted average of the nitrogen and ^1^H chemical shifts (ΔAvg_HN_), derived from Equation 1 (53)

(1) $$\Delta {\text{Avg}}_{\text{HN}}={\left[{\left(\Delta \text{HN}\right)}^{2}+{\left(\Delta \text{N}/5\right)}^{2}\right]}^{1/2}$$

where ΔHN and ΔN are the changes in the proton and nitrogen dimensions, respectively. One SD has been used as a cutoff to mark significant change.

### NMR titration studies

^15^N-labeled C_8_-, apo-, or holo-ACP in 50 mM Tris and 200 mM NaCl, pH 7.8 was titrated with LipBK135A/C169A, and chemical shift perturbations were followed in a ^15^N TROSY-HSQC experiment. ^15^N-labeled holo-ACP and octanoyl-ACP were first prepared by converting apo-ACP to C_8_-ACP using *B. subtilis* phosphopantetheinyl transferase and respective CoA. For each titration point, a ^1^H^15^N TROSY-HSQC spectra were acquired. ^1^H^15^N TROSY spectra of apo-/holo-/C_8_-*Ec*ACP were acquired at a concentration of 0.3 to 0.4 mM and then increasing concentrations of the unlabeled protein *Ec*LipBK135A/C169A was added starting from 0.25 M equivalents to 2 M equivalents sequentially and the spectra recorded after each addition. After each addition, the concentration of apo-/holo-/C_8_-*Ec*ACP was recalculated taking into account the dilution factor due to *Ec*LipBK135A/C169A addition. Thus, after each titration point, the amount of *Ec*ACP decreased and *Ec*LipBK135A/C169A increased. The concentration of ACP, as well as the enzyme stocks used for NMR studies and enzyme assays, were calculated using Bradford reagent in triplicates, and every time a new standard curve was prepared so as to minimize error.

The buffers were prepared from a single stock solution of 250 mM Tris–HCl, pH 7.8, containing 1 M NaCl stored at 4 °C, which was diluted fivefold to obtain the working concentration (50 mM Tris–HCl, pH 7.8, and 200 mM NaCl) for NMR studies.

### Binding studies using SPR

Binding strength of LipB for its substrates was determined by Autolab ESPRIT SPR spectrometer and analyzed by Autolab SPR kinetic evaluation software (Metrohm Autolab B.V.). Gold surface was activated by N-hydroxysuccinimide (0.05 M)/N-ethyl-N-(diethylaminopropyl) and carboiimide (0.2 M). LipB was immobilized on the gold surface in two channels. Unoccupied sites were blocked with 100 mM ethanolamine. After each experiment, regeneration was carried out with 50 mM NaOH. Increasing concentration of the substrate was injected on the sensor surface. Association and dissociation kinetics were monitored for 300 s and 150 s, respectively, in 20 mM sodium phosphate buffer, pH 8.0, and 100 mM NaCl. All experiments were carried out at 298 K. The SDs have been obtained from global fitting of five injections using 1:1 Langmuir model.

### Enzyme assay

Enzyme activity was performed using 5 μM LipB, 75 μM C_8_-CoA/octanoyl-ACP as the donor substrate, and 25 μM GcvH as the acceptor substrate in 50 mM Tris–HCl, 200 mM NaCl, pH 8.0, at 30 °C. The reaction components were incubated for 2 h at 30 °C and then quenched by adding the 1× SDS loading dye and stored at −20 °C overnight. The samples were loaded on a 12% SDS-PAGE gel for qualitative estimation of the conversion of GcvH or native-PAGE gel to follow breakdown of C_8_-ACP. For quantitative estimation, reverse phase Symmetry C18-5 μM Waters HPLC column was used. Twenty microliter of the enzyme assay was quenched using 0.1% TFA. The sample was injected onto the C18 Waters column. Proteins were eluted by linear gradient of 0 to 100% acetonitrile, containing 0.1% TFA. For all the assays, absorbance was observed at 220 nm. The apo/unmodified protein eluted prior to holo/C_8_-bound form. The area under the peak was integrated to determine the concentration of apo-form and holo-form. Kinetic parameters were estimated by GraphPad Prism version 7.0.

For LipT2 assay, the reaction mixture was incubated at 37 °C, and the buffer comprised 50 mM Tris–HCl, 500 mM NaCl, pH 8.0. The octanoate acceptor substrate was GcvH in all the assays.

All assays were done in duplicates, and in some cases, the error bars were even smaller than the symbols.

### Mass spectrometry

The assay samples were passed through C_4_ Zip Tips to remove salt, and the final sample was eluted in 50% acetonitrile and 0.1% TFA for analysis in Applied Biosystems 4800 MALDI-TOF spectrometer. Mass Spectrometry was used only to confirm full conversion.

### GROMACS simulations

All MD simulations were performed using GROMACS 2021.5 software, installed on a Mac, using OPLS-All Atom force field (36). *M. tuberculosis* LipB PDB file 1W66 (without the ligand or water) was used for WT protein simulations. For the LipBG77A/G78A and LipBR76A mutants, Chimera (33) was used to mutate the side chain in the PDB file, and the generated PDB file was used for simulations. The protein was solvated in a cubic box of TIP3P water molecules, based on single point charge water model (54). One Na+ in WT, as well as LipBG77A/G78A, and two Na+ in LipBR76A were used by randomly replacing solvent molecules to neutralize the protein. This resulted in a total of 41,015, 41,018, and 41,005 atoms for the WT, LipBG77A/G78A, and LipBR76A, respectively. A second energy minimization step was performed to prevent any steric clashes employing the steepest descent algorithm, until the maximum force in the system was less than 1000.0 kJmol^−1^ nm^−1^ and potential energy was negative ∼10^5^. The minimized structure was equilibrated with solvent and ions under constant number of particles (N), volume (V), and temperature (T) (NVT) and constant number of particles (N), system pressure (P) and temperature (T) (NPT) ensembles applying a positional restraining force on the heavy atoms. NVT thermal equilibration was performed using velocity-rescaling temperature coupling for 100 ps at 300 K using a modified Berendsen thermostat with a time constant of 0.1 ps. The temperature of the system reached the target value of 300 K and was stable for the rest of the equilibration. Equilibration of pressure (1 bar) was done for 100 ps under an NPT ensemble, using the Parrinello–Rahman temperature coupling, with a time constant of 2 ps. In NVT and NPT simulations, bonds to hydrogen atom were constrained using LINCS algorithm (55). Next, MD production simulation was done, for 20 ns at constant temperature (300 K) and pressure (1.0 bar) using 10,000,000 steps and integration time step of 2 fs. Electrostatic interactions were computed using a particle mesh Ewald summation algorithm (56), coulomb radius was set to 1 nm, and Van der Waals radius cut off to 1.0 nm. The command trjconv was used to center the protein in the unit cell, and a corrected trajectory was generated. RMSD/RMSF values as well as PDB files were generated from this corrected trajectory file. In all three proteins, RMSD relative to the structure present in the minimized, equilibrated system was found to level off within 20 ns.

## Data availability

Data are available from the authors upon request. Please send request to Monica Sundd, monicasundd@nii.ac.in.

## Supporting information

This article contains supporting information.

## Conflict of interest

The authors declare that they have no conflicts of interest with the contents of this article.
