# Supplementary material for: Lipoate protein ligase B primarily recognizes the C8-phosphopantetheine arm of its donor substrate and weakly binds the acyl carrier protein
Source: J Biol Chem. 2022 Jun 25;298(8):102203. doi: 10.1016/j.jbc.2022.102203 (PMC9307952; doi:10.1016/j.jbc.2022.102203)
Supplement: Supplemental Figures S1–S8 [file mmc1.pdf]

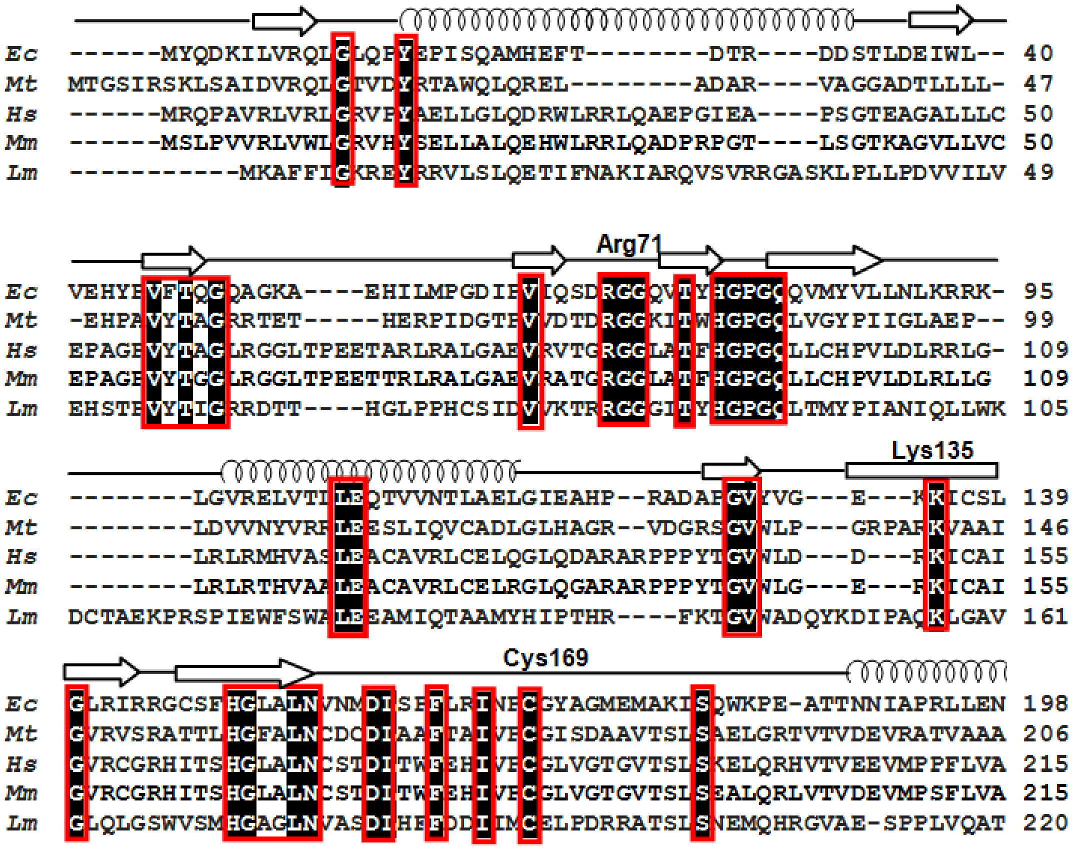

Fig. S1 Sequence comparison of *E. coli* LipB (*Ec*) with octanoyl-transferases from other sources. *Mt, Mycobacterium tuberculosis; Hs, Homo sapiens; Mm, Mus musculus; Lm, Leishmania major*. Conserved residues are shown as red boxes.

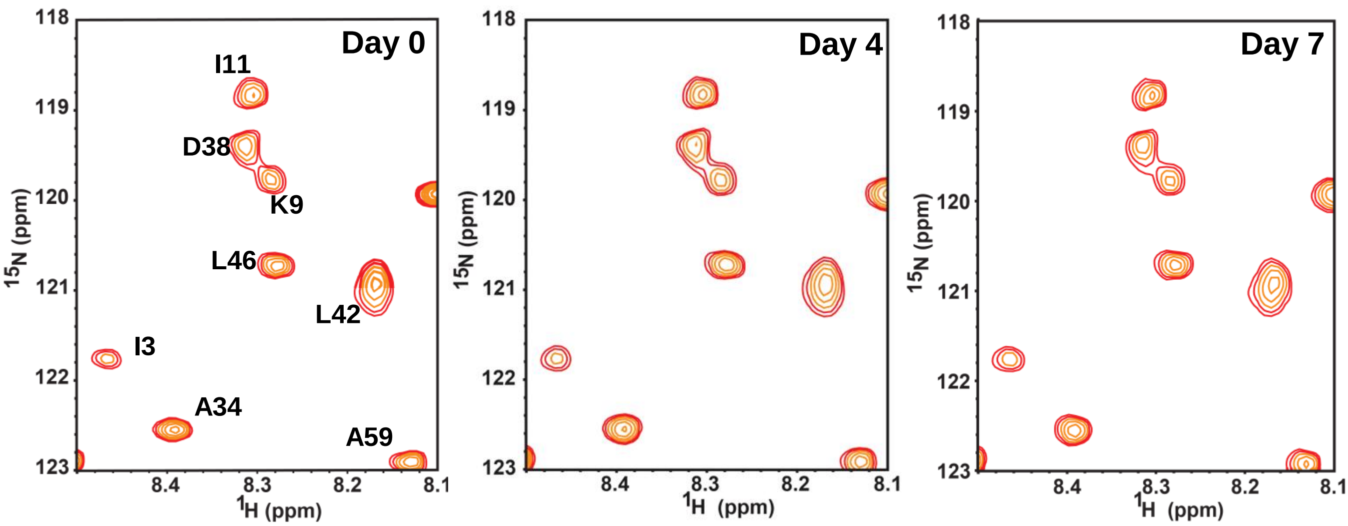

Fig. S2 *E. coli* C8-ACP hydrolysis over time. $^{1}H^{15}N$ HSQC spectra of C8-ACP on Day 0, day 4 and day 7. The spectra did not display a significant change over a period of 7 days at 298K.

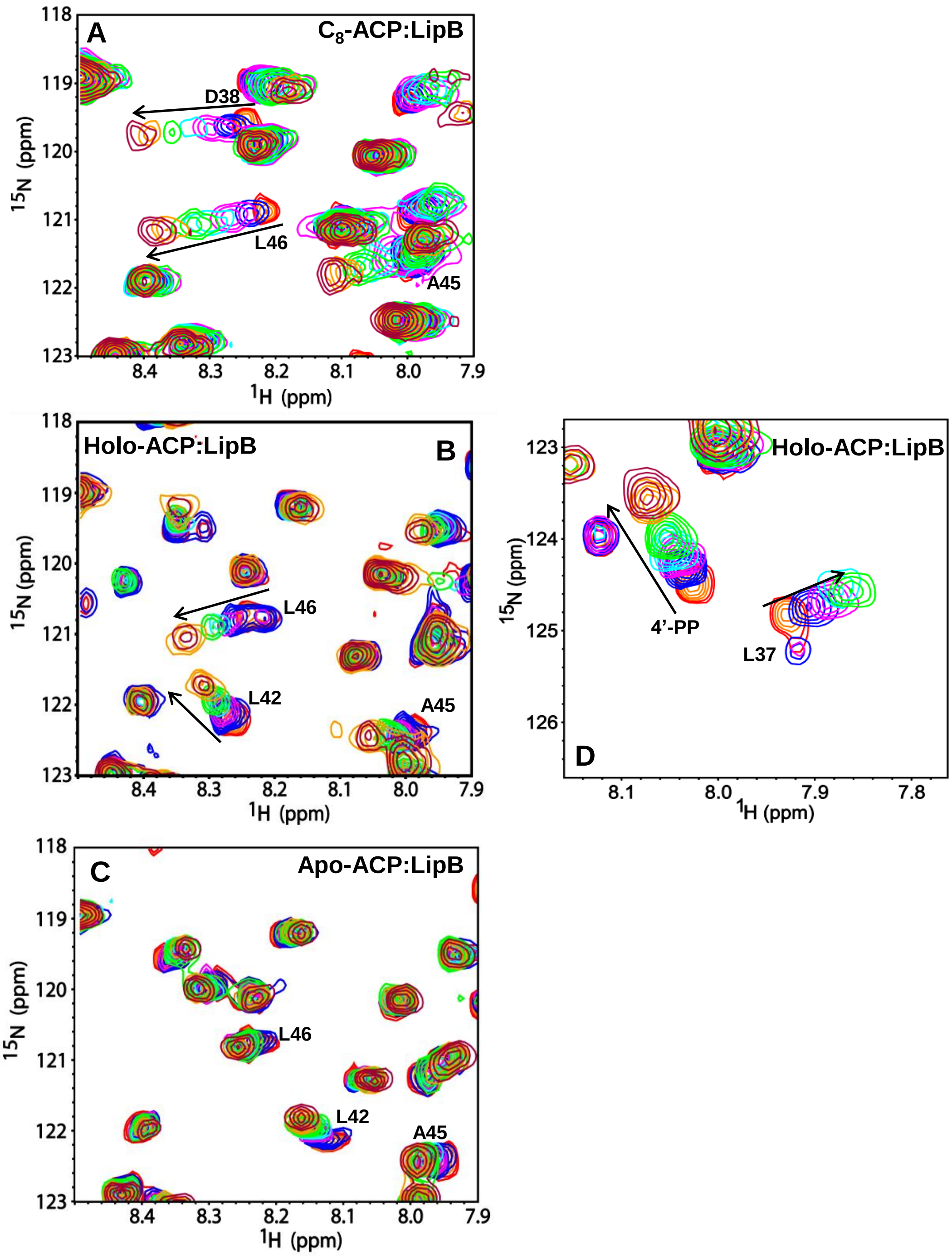

Fig. S3. Multiple overlaid $^{1}H^{15}N$ TROSY-HSQC spectra for the titration of ACP with LipBK135A/C169A. A region of the $^{1}H^{15}N$ TROSY-HSQC spectra for A) C_8-ACP, B) holo-ACP, C) apo-ACP, and D) holo-ACP 4'-PP HN peak upon titration with LipBK135A/C169A. Red peaks represent free ACP, blue: 1:0.25, magenta 1:0.5, cyan 1:0,75, green 1:1, orange 1:1.5 and maroon 1:2 ACP: LipBK135A/C169A molar ratio. Residues that display significant perturbations are labeled.

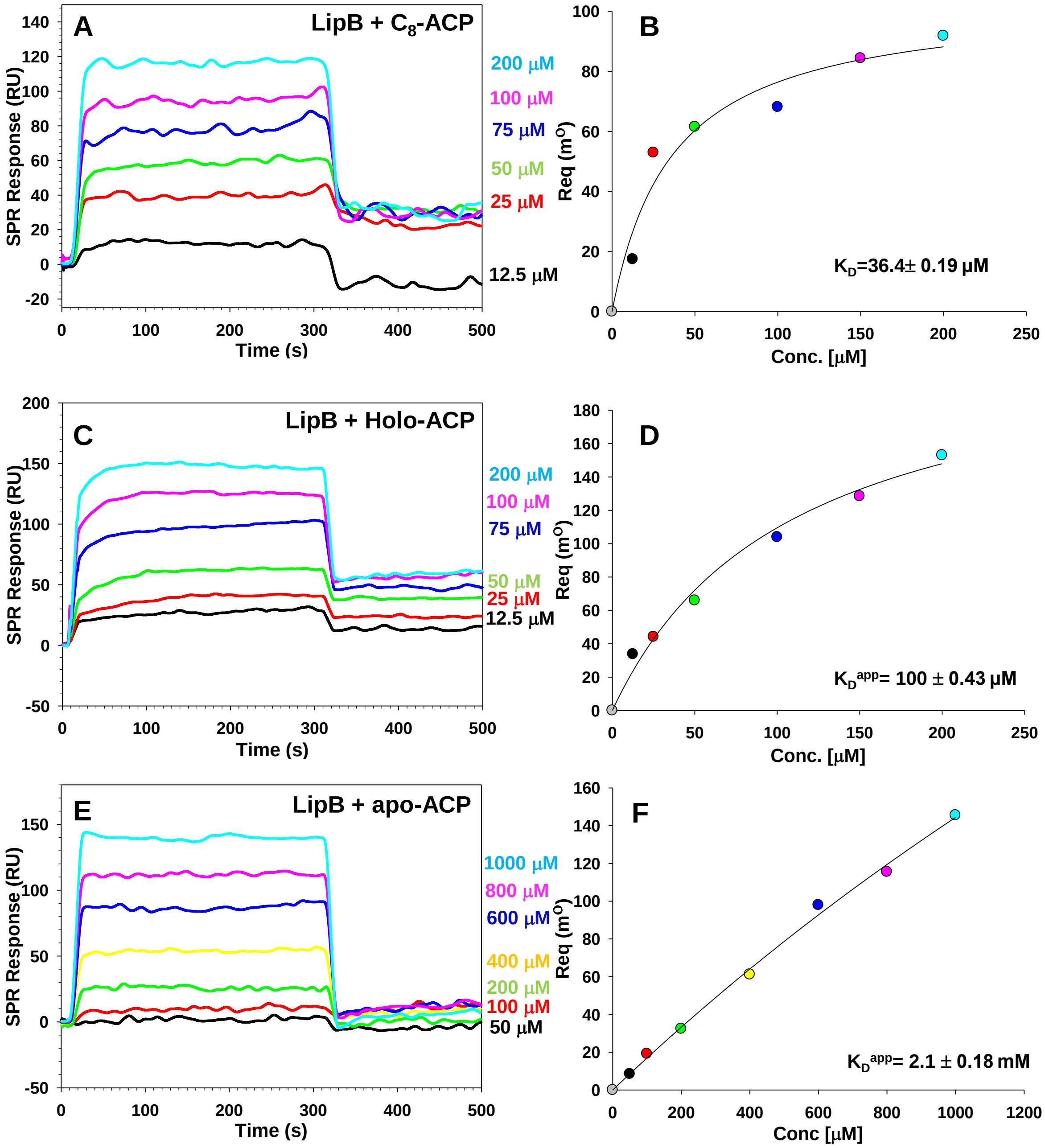

Fig. S4. Surface Plasmon Resonance measurements for the interaction of LipB with ACP. SPR sensorgrams for the binding of immobilized LipBK135A/C169A with A) C_8-ACP, C) Holo-ACP, and E) apo-ACP. The color of different sensorgrams correspond to the concentration mentioned on the right side of the figure, used to pass over the immobilized sample. A plot of maximum response reached at equilibrium (Req) for each concentration of ligand used in the sensorgram for B) C_8-ACP, D) holo-ACP, and F) apo-ACP. Due to incomplete saturation of holo- and apo-ACP binding curves, apparent binding affinity has been reported for the SPR measurements.

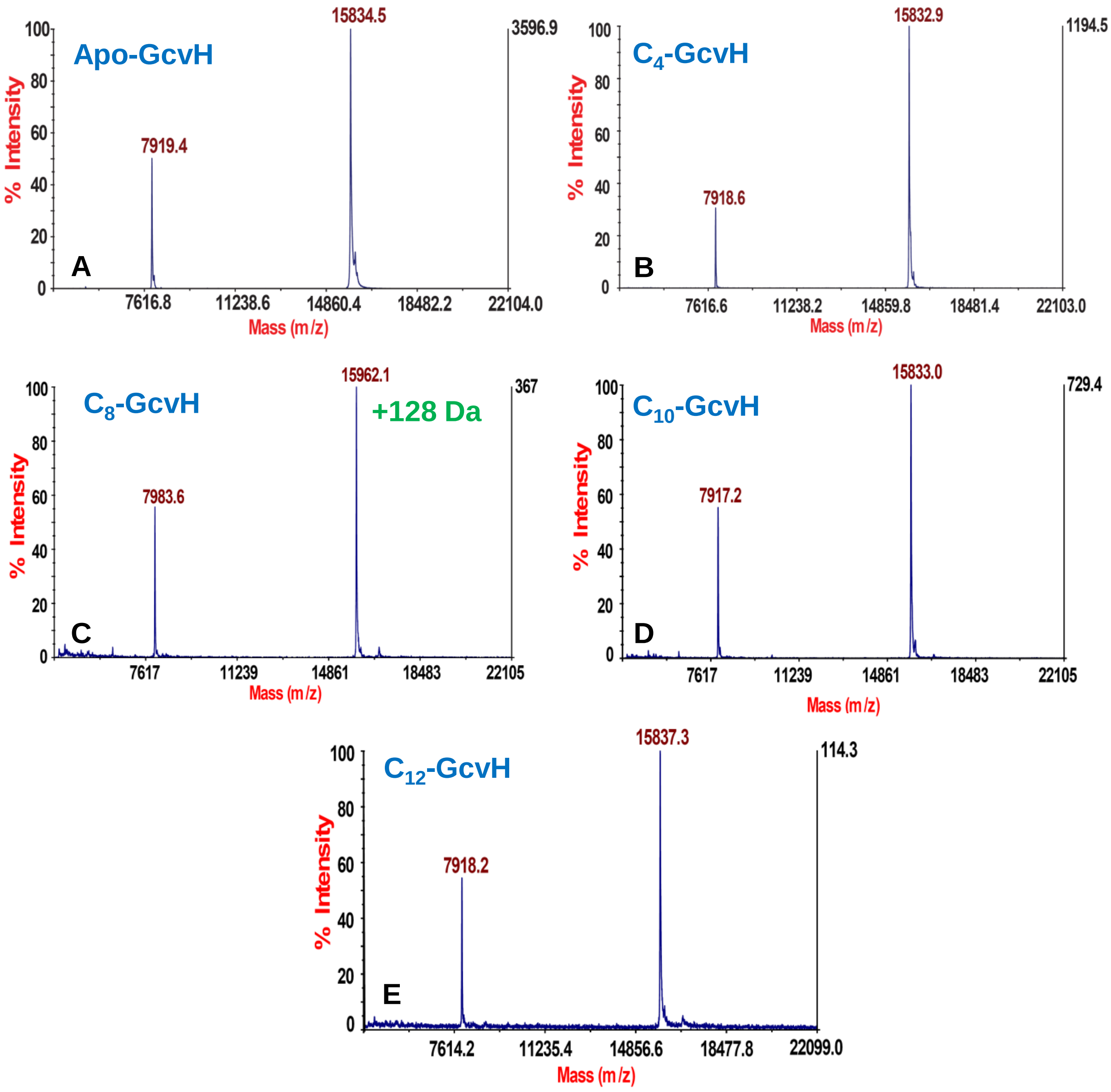

Fig. S5. Mass Spectrometry confirms that LipB can transfer octanoyl- chain from C_8-CoA to GcvH. MALDI-TOF mass spectra for A) apo-GcvH used as a control (monoisotopic mass 15834Da). GcvH in the assay performed using B) C_4-CoA, C) C_8-CoA, and D) C_{10}-CoA, and E) C_{12}-CoA as acyl- chain donors. A 128 kDa increase in mass was observed when C_8-CoA was used as a donor, nearly equivalent to the mass of the C_8-chain (127 kDa).

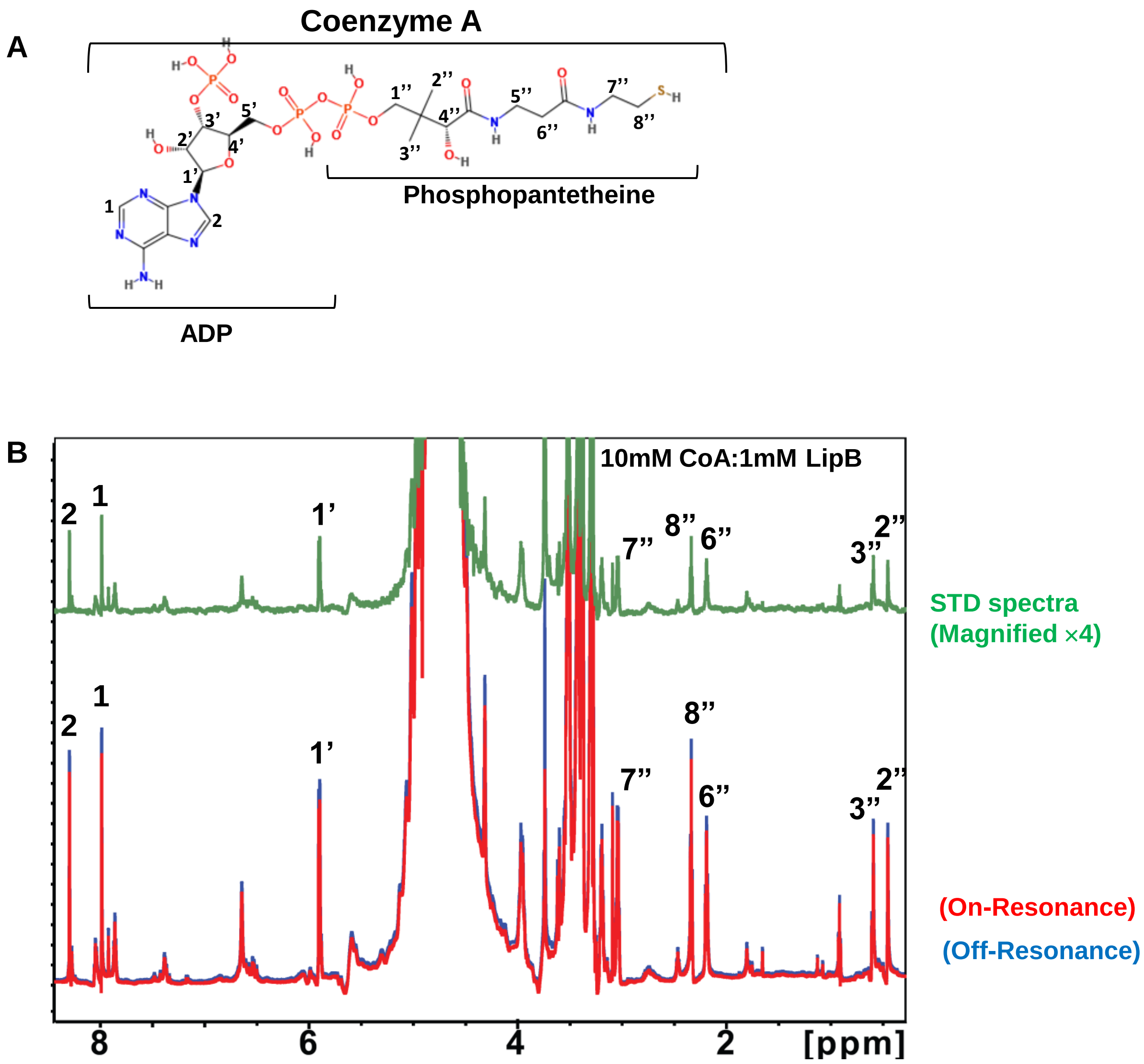

Fig. S6. ^{1}H Saturation transfer difference (STD) spectra for the interaction of CoA with LipBK135A/C169A. A) Chemical structure of CoA. B) ^{1}H STD spectra for the LipB:CoA interaction. Red spectra represents on-resonance, blue represents off-resonance, and green spectra is the STD difference spectra.

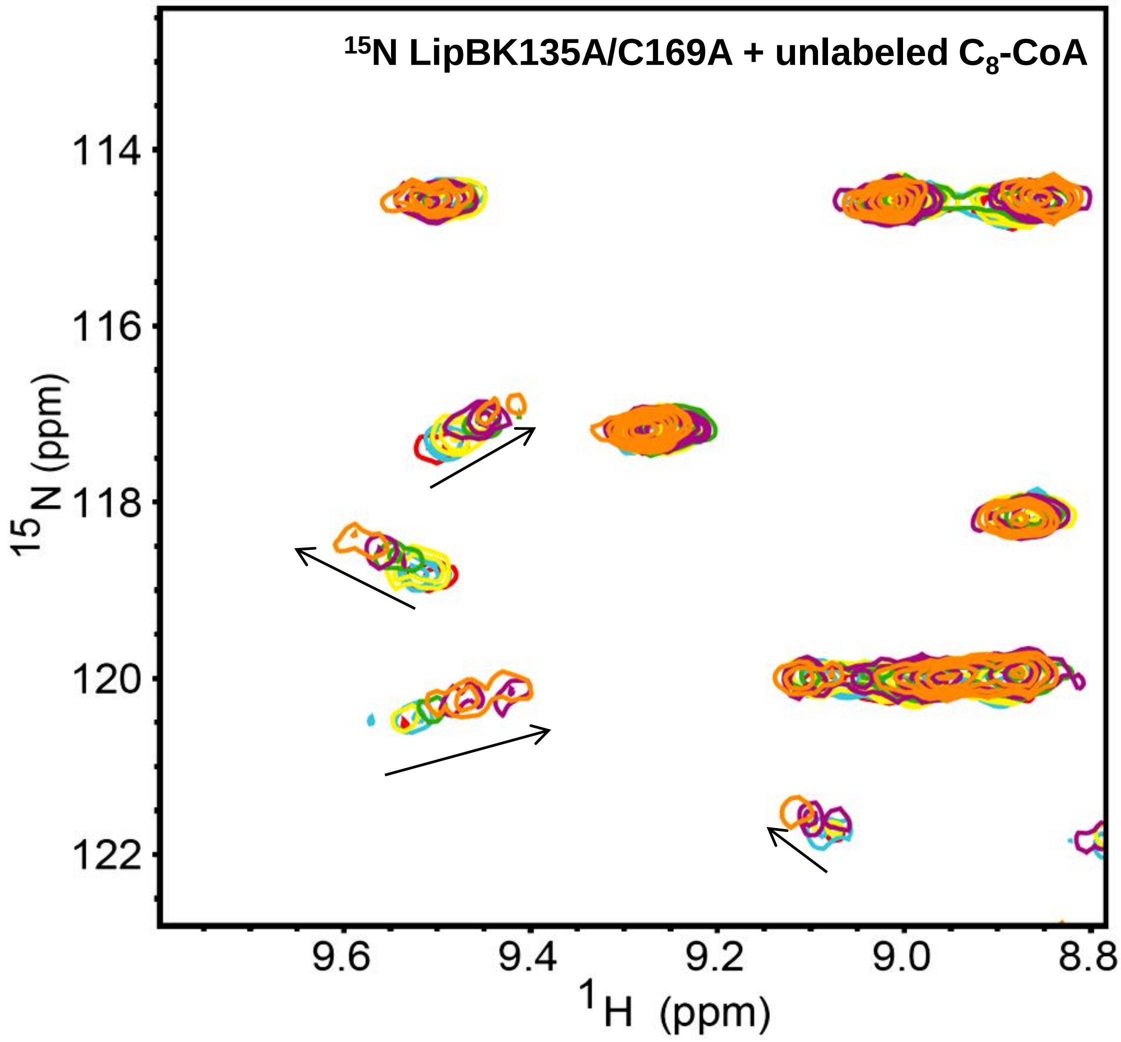

Fig. S7 Interaction of $^{1}H^{15}N$ LipBK135A/C169A with C_8-CoA. $^{1}H^{15}N$ TROSY-HSQC spectra of LipBK135A/C169A in 50mM Tris, 200mM NaCl, pH 7.8 with increasing C_8-CoA concentrations; 1:0 molar ratio (red), 1:0.2 (cyan), 1:0.4 (yellow), 1:0.8 (green), 1:1 (magenta), 1:2 (orange).

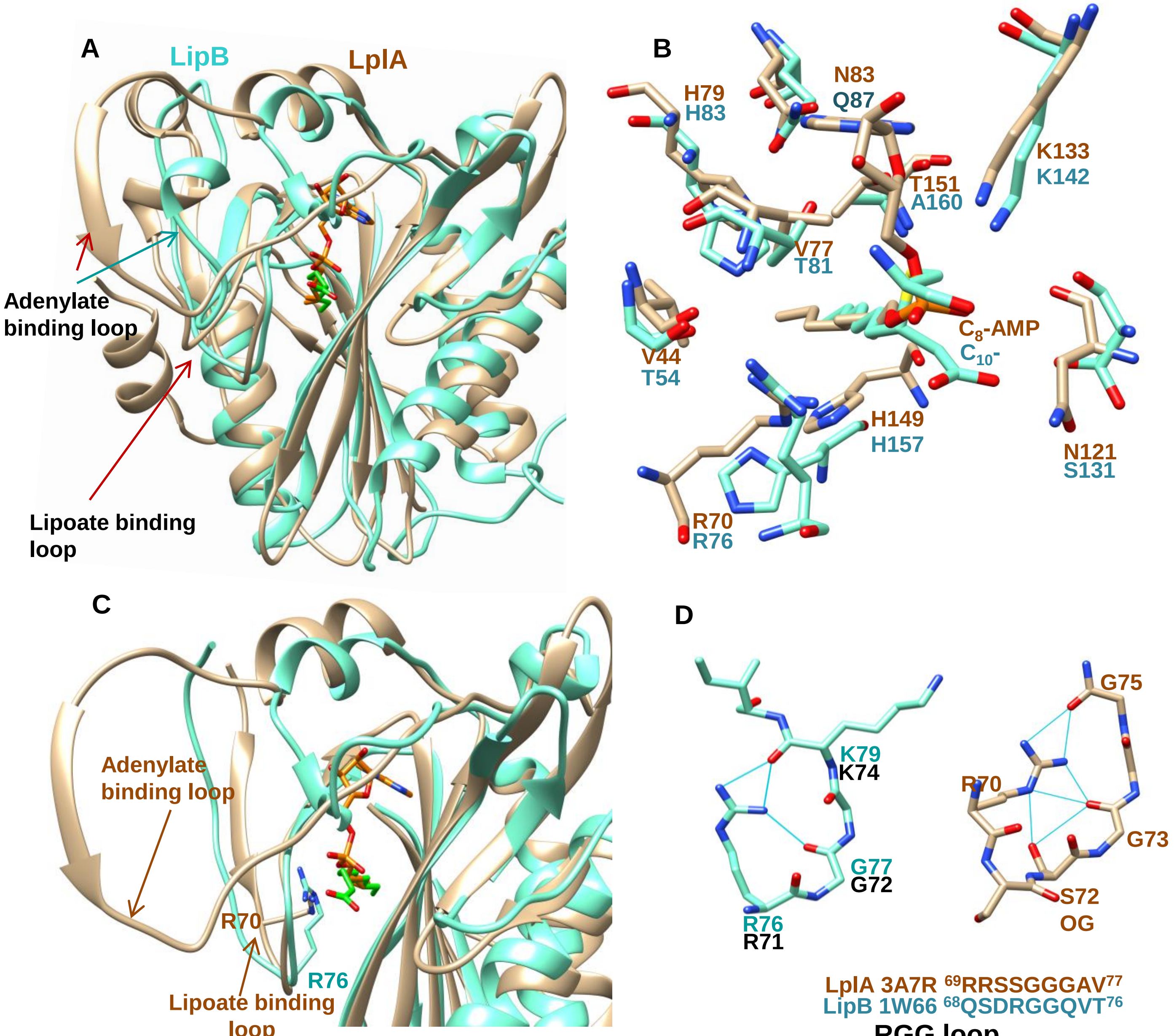

Fig. S8 *M. tuberculosis* LipB structure comparison with *E. coli* LplA structure. A) Ribbon representation of *M. tuberculosis* LipB (PDB 1W66, colored cyan) superimposed on the *E. coli* LplA catalytic domain structure (PDB 3A7R, colored brown). B) LplA-AMP interaction. LplA side chains that interact with lipoyl-5-AMP are colored light brown. The corresponding side chains of *M. tuberculosis* LipB are colored cyan. C) The lipoate binding loop of LplA overlaid on the RGG loop of *Mt*LipB (PDB 1W66). D) Side chain to backbone hydrogen bonds of Arg 76 side chain with the backbone carbonyls of other residues of the RGG loop of *Mt*LipB (PDB 1W66) are shown. These interactions are similar to the side chain interactions of Arg 69 in the lipoate binding loop of LplA (PDB 3A7R).
